# ﻿*Sinocyclocheiluslongicornus* (Cypriniformes, Cyprinidae), a new species of microphthalmic hypogean fish from Guizhou, Southwest China

**DOI:** 10.3897/zookeys.1141.91501

**Published:** 2023-01-17

**Authors:** Cheng Xu, Tao Luo, Jia-Jun Zhou, Li Wu, Xin-Rui Zhao, Hong-Fu Yang, Ning Xiao, Jiang Zhou

**Affiliations:** 1 School of Karst Science, Guizhou Normal University, Guiyang 550001, Guizhou, China; 2 School of Life Sciences, Guizhou Normal University, Guiyang 550025, Guizhou, China; 3 Zhejiang Forest Resource Monitoring Center, Hangzhou 310020, Zhejiang, China; 4 Zhejiang Forestry Survey Planning and Design Company Limited, Hangzhou 310020, Zhejiang, China; 5 Fishery Administration of Qiubei County, Qiubei 663200, Yunnan, China; 6 Guiyang Healthcare Vocational University, Guiyang 550081, Guizhou, China

**Keywords:** cave fish, morphology, taxonomy, phylogeny

## Abstract

*Sinocyclocheiluslongicornus***sp. nov.** is described from the Pearl River basin in Hongguo Town, Panzhou City, Guizhou Province, Southwest China. Based on the presence of the long horn-like structure on the back of the head, *Sinocyclocheiluslongicornus***sp. nov.** is assigned to the *Sinocyclocheilusangularis* species group. *Sinocyclocheiluslongicornus***sp. nov.** is distinguished from its congeners by a combination of morphological characters: (1) presence of a single, relatively long horn-like structure on the back of the head; (2) pigmentation absent; (3) reduced eyes; (4) dorsal-fin rays, ii, 7; (5) pectoral-fin rays, i, 13; (6) anal-fin rays, iii, 5; (7) pelvic-fin rays, i, 7; (8) lateral line pores 38–49; (9) gill rakers well developed, nine on first gill arch; and (10) tip of adpressed pelvic fin not reaching anus.

## ﻿Introduction

The golden-line fish genus *Sinocyclocheilus* Fang, 1936, is endemic to China, and is mainly distributed in the karst areas of Southwest China, including Guangxi, Guizhou, Yunnan, and Hubei provinces (Zhao and Zhang 2009; Jiang et al. 2019). The narrow distribution, morphological similarities, and morphological adaptations to cave environments, such as the degeneration or loss of eyes and body scales, have made classification of the genus difficult and often controversial (Chu and Cui 1985; Shan and Yue 1994; Wang et al. 1995; Wang and Chen 1998; Wang et al. 1999; Wang and Chen 2000; Xiao et al. 2005; Mao et al. 2021, 2022; Wen et al. 2022). A phylogenetic study based on the mitochondrial cytochrome b gene (Cyt *b*) showed that all members of *Sinocyclocheilus* clustered as a monophyletic group, divided into four species groups, namely the *S.jii*, *S.angularis*, *S.cyphotergous*, and *S.tingi* groups (Zhao and Zhang 2009). However, phylogenetic studies based on restriction site–associated DNA sequencing and mitochondrial genome reconstruction suggest that the *S.angularis* and *S.cyphotergous* species groups are not monophyletic (Xiang 2014; Liu 2018; Mao et al. 2021, 2022; Wen et al. 2022). *Sinocyclocheilus* comprises 76 valid species, of which 71 species are grouped into five species groups (Table 1).

**Table 1.** List of 76 currently recognized species of the genus *Sinocyclocheilus* endemic to China and references. Recognized species modified from Jiang et al. (2019).

ID	Species	Species group	Province	River	Reference
1	*S.altishoulderus* (Li & Lan, 1992)	*S.angularis* group	Guangxi	Hongshuihe River	Li and Lan 1992
2	*S.anatirostris* Lin & Luo, 1986	*S.angularis* group	Guangxi	Hongshuihe River	Lin and Luo 1986
3	*S.angularis* Zheng & Wang, 1990	*S.angularis* group	Guizhou	Nanpanjiang River	Zheng and Wang 1990
4	*S.aquihornes* Li & Yang, 2007	*S.angularis* group	Yunnan	Nanpanjiang River	Li et al. 2007
5	*S.bicornutus* Wang & Liao, 1997	*S.angularis* group	Guizhou	Beipanjiang River	Wang and Liao 1997
6	*S.brevibarbatus* Zhao, Lan & Zhang, 2009	*S.angularis* group	Guangxi	Hongshuihe River	Zhao et al. 2009
7	*S.broadihornes* Li & Mao, 2007	*S.angularis* group	Yunnan	Nanpanjiang River	Li and Mao 2007
8	*S.convexiforeheadus* Li, Yang & Li, 2017	*S.angularis* group	Yunnan	Nanpanjiang River	Yang et al. 2017
9	*S.hyalinus* Chen & Yang, 1994	*S.angularis* group	Yunnan	Nanpanjiang River	Chen et al. 1994
10	*S.jiuxuensis* Li & Lan, 2003	*S.angularis* group	Guangxi	Hongshuihe River	Li et al. 2003c
11	*S.flexuosdorsalis* Zhu & Zhu, 2012	*S.angularis* group	Guangxi	Hongshuihe River	Zhu and Zhu 2012
12	*S.furcodorsalis* Chen, Yang & Lan, 1997	*S.angularis* group	Guangxi	Hongshuihe River	Chen et al. 1997
13	*S.mashanensis* Wu, Liao & Li, 2010	*S.angularis* group	Guangxi	Hongshuihe River	Wu et al. 2010
14	*S.rhinocerous* Li & Tao, 1994	*S.angularis* group	Yunnan	Nanpanjiang River	Li and Tao 1994
15	*S.simengensis* Li, Wu, Li & Lan, 2018	*S.angularis* group	Guangxi	Hongshuihe River	Wu et al. 2018
16	*S.tianeensis* Li, Xiao & Luo, 2003	*S.angularis* group	Guangxi	Hongshuihe River	Li et al. 2003d
17	*S.tianlinensis* Zhou, Zhang, He & Zhou, 2004	*S.angularis* group	Guangxi	Nanpanjiang River	Zhou et al. 2004
18	*S.tileihornes* Mao, Lu & Li, 2003	*S.angularis* group	Yunnan	Nanpanjiang River	Mao et al. 2003
19	*S.zhenfengensis* Liu, Deng, Ma, Xiao & Zhou, 2018	*S.angularis* group	Guizhou	Beipanjiang River	Liu et al. 2018
20	*S.anshuiensis* Gan, Wu, Wei & Yang, 2013	*S.microphthalmus* group	Guangxi	Hongshuihe River	Gan et al. 2013
21	*S.microphthalmus* Li, 1989	*S.microphthalmus* group	Guangxi	Hongshuihe River	Li 1989
22	*S.aluensis* Li & Xiao, 2005	*S.tingi* group	Yunnan	Nanpanjiang River	Li et al. 2005; Zhao and Zhang 2013
23	*S.angustiporus* Zheng & Xie, 1985	*S.tingi* group	Guizhou; Yunnan	Beipanjiang River; Nanpanjiang River	Zheng and Xie 1985
24	*S.anophthalmus* Chen & Chu, 1988	*S.tingi* group	Yunnan	Nanpanjiang River	Chen et al. 1988a Zhao and Zhang 2009
25	*S.grahami* (Regan, 1904)	*S.tingi* group	Yunnan	Jinshajiang River	Regan 1904; Zhao and Zhang 2009
26	*S.guishanensis* Li, 2003	*S.tingi* group	Yunnan	Nanpanjiang River	Li et al. 2003a
27	*S.huaningensis* Li, 1998	*S.tingi* group	Yunnan	Nanpanjiang River	Li et al. 1998
28	*S.huizeensis* Cheng, Pan, Chen, Li, Ma & Yang, 2015	*S.tingi* group	Yunnan	Niulanjiang River	Cheng et al. 2015
29	*S.bannaensis* Li, Li & Chen, 2019	*S.tingi* group	Yunnan	Luosuojiang River	Li et al. 2019
30	*S.maculatus* Li, 2000	*S.tingi* group	Yunnan	Nanpanjiang River	Zhao and Zhang 2009
31	*S.maitianheensis* Li,1992	*S.tingi* group	Yunnan	Nanpanjiang River	Li 1992
32	*S.malacopterus* Chu & Cui, 1985	*S.tingi* group	Yunnan	Nanpanjiang River	Chu and Cui 1985
33	*S.longifinus* Li, 1998	*S.tingi* group	Yunnan	Nanpanjiang River	Li et al. 1998
34	*S.longshanensis* Li & Wu, 2018	*S.tingi* group	Yunnan	Nanpanjiang River	Li et al. 2018
35	*S.macrocephalus* Li,1985	*S.tingi* group	Yunnan	Nanpanjiang River	Li 1985
36	*S.lateristriatus* Li,1992	*S.tingi* group	Yunnan	Nanpanjiang River	Li 1992
37	*S.purpureus* Li, 1985	*S.tingi* group	Yunnan	Nanpanjiang River	Li 1985
38	*S.qiubeiensis* Li, 2002	*S.tingi* group	Yunnan	Nanpanjiang River	Li et al. 2002b
39	*S.qujingensis* Li, Mao & Lu, 2002	*S.tingi* group	Yunnan	Nanpanjiang River	Li et al. 2002c
40	*S.robustus* Chen & Zhao, 1988	*S.tingi* group	Guizhou	Nanpanjiang River	Chen et al. 1988b
41	*S.wumengshanensis* Li, Mao, Lu & Yan, 2003	*S.tingi* group	Yunnan	Niulanjiang River	Li et al. 2003a
42	*S.xichouensis* Pan, Li, Yang & Chen, 2013	*S.tingi* group	Yunnan	Panlonghe River	Pan et al. 2013
43	*S.tingi* Fang, 1936	*S.tingi* group	Yunnan	Nanpanjiang River	Fang, 1936; Zhao and Zhang 2009
44	*S.yangzongensis* Chu & Chen, 1977	*S.tingi* group	Yunnan	Nanpanjiang River	Wu 1977; Zhao and Zhang 2009
45	*S.yimenensis* Li & Xiao, 2005	*S.tingi* group	Yunnan	Yuanjiang River	Li et al. 2005
46	*S.oxycephalus* Li, 1985	*S.tingi* group	Yunnan	Nanpanjiang River	Li 1985
47	*S.brevis* Lan & Chen, 1992	*S.cyphotergous* group	Guangxi	Liujiang River	Chen and Lan 1992
48	*S.cyphotergous* (Dai, 1988)	*S.cyphotergous* group	Guizhou	Hongshuihe River	Dai 1988; Huang et al. 2017
49	*S.donglanensis* Zhao, Watanabe & Zhang, 2006	*S.cyphotergous* group	Guangxi	Hongshuihe River	Zhao et al. 2006
50	*S.dongtangensis* Zhou, Liu & Wang, 2011	*S.cyphotergous* group	Guizhou	Liujiang River	Zhou et al. 2011
51	*S.huanjiangensis* Wu, Gan & Li, 2010	*S.cyphotergous* group	Guangxi	Liujiang River	Wu et al. 2010
52	*S.hugeibarbus* Li, Ran & Chen, 2003	*S.cyphotergous* group	Guizhou	Liujiang River	Li et al. 2003b
53	*S.gracilicaudatus* Zhao & Zhang, 2014	*S.cyphotergous* group	Guangxi	Liujiang River	Wang et al. 2014
54	*S.lingyunensis* Li, Xiao & Lu, 2000	*S.cyphotergous* group	Guangxi	Hongshuihe River	Li et al. 2000
55	*S.longibarbatus* Wang & Chen, 1989	*S.cyphotergous* group	Guizhou; Guangxi	Liujiang River	Wang and Chen 1989
56	*S.luopingensis* Li & Tao, 2002	*S.cyphotergous* group	Yunnan	Nanpanjiang River	Li et al. 2002a
57	*S.macrolepis* Wang & Chen, 1989	*S.cyphotergous* group	Guizhou; Guangxi	Liujiang River	Wang and Chen 1989
58	*S.macrophthalmus* Zhang & Zhao, 2001	*S.cyphotergous* group	Guangxi	Hongshuihe River	Zhang and Zhao 2001
59	*S.macroscalus* Li, 1992	*S.tingi* group	Yunnan	Nanpanjiang River	Li 1992
60	*S.multipunctatus* (Pellegrin, 1931)	*S.cyphotergous* group	Guizhou; Guangxi	Wujiang River; Liujiang River; Hongshuihe River	Pellegrin 1931; Zhao and Zhang 2009
61	*S.punctatus* Lan & Yang, 2017	*S.cyphotergous* group	Guizhou; Guangxi	Liujiang River; Hongshuihe River	Lan et al. 2017
62	*S.ronganensis* Luo, Huang & Wen, 2016	*S.cyphotergous* group	Guangxi	Liujiang River	Luo et al. 2016
63	*S.xunlensis* Lan, Zhan & Zhang, 2004	*S.cyphotergous* group	Guangxi	Liujiang River	Lan et al. 2004
64	*S.yaolanensis* Zhou, Li & Hou, 2009	*S.cyphotergous* group	Guizhou	Liujiang River	Zhou et al. 2009
65	*S.yishanensis* Li & Lan, 1992	*S.cyphotergous* group	Guangxi	Liujiang River	Li and Lan 1992
66	*S.sanxiaensis* Jiang, Li, Yang & Chang, 2019	*S.cyphotergous* group	Hubei	Yangtze River	Jiang et al. 2019
67	*S.brevifinus* Li, Li & Mayden, 2014	*S.jii* group	Guangxi	Hejiang River	Li et al. 2014
68	*S.guanyangensis* Chen, Peng & Zhang, 2016	*S.jii* group	Guangxi	Guijiang River	Chen et al. 2016
69	*S.guilinensis* Ji, 1985	*S.jii* group	Guangxi	Guijiang River	Zhou 1985; Zhao and Zhang 2009
70	*S.huangtianensis* Zhu, Zhu & Lan, 2011	*S.jii* group	Guangxi	Hejiang River	Zhu et al. 2011
71	*S.jii* Zhang & Dai, 1992	*S.jii* group	Guangxi	Guijiang River	Zhang and Dai 1992
72	*S.gracilis* Li & Li, 2014	No assignment	Guangxi	Guijiang River	Li and Li 2014
73	*S.pingshanensis* Li, Li, Lan & Wu, 2018	No assignment	Guangxi	Liujiang River	Wu et al. 2018
74	*S.wenshanensis* Li,Yang, Li & Chen, 2018	No assignment	Yunnan	Panlonghe River	Yang et al. 2018
75	*S.wui* Li & An, 2013	No assignment	Yunnan	Mingyihe River	Li and An 2013
76	*S.luolouensis* Lan, 2013	No assignment	Guangxi	Hongshuihe River	Lan et al. 2013

**Figure 1.**
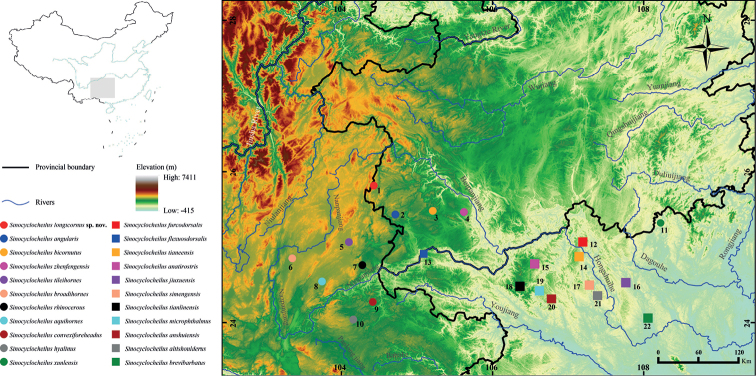
Sampling collection localities and distribution of the *Sinocyclocheiluslongicornus* sp. nov. and 21 species of the *S.angularis* and *S.microphthalmus* species groups of the genus *Sinocyclocheilus* in Southwest China. **1.** Hongguo Town, Panzhou City, Guizhou Province. **2.** Baotian Town, Panzhou City, Guizhou Province. **3.** Xinlongchang Town, Xingren City, Guizhou Province. **4.** Zhexiang Town, Zhenfeng County, Guizhou Province. **7**. Huancheng Town, Luoping County, Yunnan Province. 5–6, 8–22 is detailed in Suppl. material 1. The maps are from Standard Map Service website (http://bzdt.ch.mnr.gov.cn/index.html).

Species of *Sinocyclocheilus* have variably developed eyes and horn-like structures on the back of the head. Eye morphology includes normal, microphthalmic, and anophthalmic conditions (Mao et al. 2021). Normal-eyed and microphthalmic species are distributed from eastern Guangxi through southern Guizhou to eastern Yunnan, and eyeless species are mainly distributed in the Hongshuihe river basin in northern Guangxi and the Nanpanjiang river basin in eastern Yunnan (Mao et al. 2021). It may be absent, short, long, or single and forked. The horn-like structure is present mainly in species of the *S.angularis* and *S.microphthalmus* species groups (Zhao and Zhang 2009; Mao et al. 2021; Wen et al. 2022). These horned species are distributed in the Nanpanjiang, Beipanjiang, and Hongshuihe river basins of the upper Pearl River (Fig. 1).

We collected specimens of a horned, scaleless, and unpigmented species of *Sinocyclocheilus* in a completely dark cave in southwestern Guizhou Province in China. Molecular phylogenetic analyses and morphological comparisons showed that these specimens represented an undescribed species of *Sinocyclocheilus*. Here, we provide the formal description of that species as *Sinocyclocheiluslongicornus* sp. nov.

## ﻿Materials and methods

### ﻿Specimen sampling

During a cavefish diversity survey in southern China in May 2021, 32 specimens of the genus *Sinocyclocheilus* were collected in southwestern Guizhou Province. Among these, 15 specimens represented an undescribed species, subject of this, paper from Hongguo Town in Panzhou City; seven were *S.angularis* from Baotian Town in Panzhou; two were *S.bicornutus* from Xiashan Town in Xingren City; and eight were *S.zhenfengensis* from Zhexiang Town in Zhenfeng County (Fig. 1). Gill muscle tissues used for molecular analysis were preserved in 95% alcohol at −20 °C. All specimens were fixed in 10% buffered formalin and later transferred to 75% ethanol for long term preservation. All specimens were deposited in Guizhou Normal University, Guiyang City, Guizhou Province, China.

### ﻿DNA Extraction, PCR amplification, and sequencing

Genomic DNA was extracted from muscle tissues using a DNA extraction kit from Tiangen Biotech Co., Ltd. (Beijing, China). Because the most used molecular markers in *Sinocyclocheilus* are fragments of the mitochondrial cytochrome b (Cyt *b*) and NADH dehydrogenase subunit 4 (*ND4*) genes, we selected these fragments for amplification and sequencing. Primers used for *Cyt b* were L14737 (5’-CCACCGTTGTTAATTCAACTAC-3’) and H15915 (5’-CTCCGATCTCCGGATTACAAGAC-3’), following Xiao et al. (2005). Primers used for *ND4* were L11264 (5’-ACGGGACTGAGCGATTAC-3’) and H12346 (5’-TCATCATATTGGGTTAG-3’), following Xiao et al. (2005). PCR ampliﬁcations were performed in a 25-μl reaction volume with the following cycling conditions: an initial denaturing step at 95 °C for 3 min; 35 cycles of denaturing at 94 °C for 50 s, annealing at 52 °C (for Cyt *b* and *ND4*) for 1 min and extension at 72 °C for 1 min, and a ﬁnal extension step of 72 °C for 10 min. The PCR products were sequenced on an ABI Prism 3730 automated DNA sequencer at Chengdu TSING KE Biological Technology Co. Ltd. (Chengdu, China). All sequences were deposited in GenBank (Table 2).

**Table 2.** Localities, voucher information, and GenBank numbers for all samples used.

ID	Species	Locality (* type localities)	Voucher number	GenBank accession No.	
				Cyt *b*	*ND4*
1	*Sinocyclocheilushuizeensis*	Leye Town, Huize County, Yunnan, China	hrfri2018046	MH982229	MH982229
2	*Sinocyclocheilusqiubeiensis*	Songming, Yunnan, China	IHB:2006624	EU366188	EU366182
3	*Sinocyclocheilusyimenensis*	Yimen, Yunnan, China	IHB:2006646	EU366191	EU366180
4	*Sinocyclocheilusgrahami*	Haikou, Kunming City, Yunnan, China	–	GQ148557	GQ148557
5	*Sinocyclocheilustingi*	Fuxian Lake, Yunnan, China	YNUST201406180002	MG323567	MG323567
6	*Sinocyclocheiluswumengshanensis*	Xuanwei County, Yunnan, China	YNUSM20160817008	MG021442	MG021442
7	*Sinocyclocheilusanophthalmus*	Jiuxiang, Yiliang County, Yunnan, China	XH3001	AY854715	AY854772
8	*Sinocyclocheilusmaculatus*	Yiliang, Yunnan, China	IHB:2006632	EU366193	EU366183
9	*Sinocyclocheilusmaitianheensis*	Jiuxiang, Yiliang County, Yunnan, China	XH2301	AY854710	AY854767
10	*Sinocyclocheiluslateristriatus*	Maojiachong, Zhanyi County, Yunnan	XH1102	AY854703	AY854760
11	*Sinocyclocheilusqujingensis*	Huize County, Yunnan, China	hrfri2018044	MH937706	MH937706
12	*Sinocyclocheilusguishanensis*	Guishan, Shilin County, Yunnan, China	XH5401	AY854722	AY854779
13	*Sinocyclocheilushuaningensis*	Huaning County, Yunnan, China	XH3701	AY854718	AY854775
14	*Sinocyclocheilusoxycephalus*	Heilongtan, Shilin County, Yunnan, China	XH0201	AY854685	AY854742
15	*Sinocyclocheilusmacrocephalus*	Heilongtan, Shilin County, Yunnan	XH0103	AY854683	AY854740
16	*Sinocyclocheilusmalacopterus*	Wulonghe, Shizong County, Yunnan, China	XH0901	AY854697	AY854754
17	*Sinocyclocheiluspurpureus*	Luoping County, Yunnan, China	IHB:2006638	EU366189	EU366178
18	*Sinocyclocheilusangustiporus*	Xinlongchnag Town, Xingren City, Guizhou, China	GZNU20210322002	MZ636515	MZ636515
19	*Sinocyclocheilusyangzongensis*	Yangzonghai Lake, Yunnan, China	XH6101	AY854725	AY854782
20	*Sinocyclocheilusmultipunctatus*	Huishui County, Guizhou, China	–	MG026730	MG026730
21	*Sinocyclocheilussanxiaensis*	Guojiaba Town, Zigui County, Hubei, China*	KNHM 2019000001	MN106258	–
22	*Sinocyclocheiluscyphotergous*	Dongdang township, Luodian County, Guizhou, China*	GZNU20150819010	MW024370	MW024370
23	*Sinocyclocheiluspunctatus*	Dongtang Township, Libo County, Guizhou, China	GZNU20150811002	MW014318	MW014318
24	*Sinocyclocheilusmacrolepis*	Nandan County, Guangxi, China	XH8201	AY854729	AY854786
25	*Sinocyclocheilusbrevis*	–	GX0155	MT373105	MW548424
26	*Sinocyclocheilushuanjiangensis*	–	GX0124	MT373103	MW548429
27	*Sinocyclocheiluslongibarbatus*	Dongtang Township, Libo County, Guizhou, China*	GZNU20150809004	MW024372	MW024372
28	*Sinocyclocheilusxunlensis*	Huanjiang, Guangxi, China	IHB:04050268	EU366187	EU366184
29	*Sinocyclocheilusdonglanensis*	Hongshuihe River, Donglan County, Guangxi, China	CA139	AB196440	MW548425
30	*Sinocyclocheiluslingyunensis*	Shadong, Lingyun County, Guangxi, China	XH0502	AY854691	AY854748
31	*Sinocyclocheilushugeibarbus*	Xiaoqikong Town, Libo County, Guizhou, China*	GZNU20150120005	MW014319	MW014319
32	*Sinocyclocheilusmacrophthalmus*	Xiaao, Duan County, Guangxi, China	XH8401	AY854733	AY854790
33	*Sinocyclocheilusyishanensis*	Liujiang County, Guangxi, China	–	MK387704	MK387704
34	*Sinocyclocheilusronganensis*	Rong’an County, Guangxi, China	–	KX778473	KX778473
35	*Sinocyclocheilusfurcodorsalis*	Tian’e County, Guangxi, China	–	GU589570	GU589570
36	*Sinocyclocheilustianlinensis*	–	GX0087-L17-16	MT373102	MW548431
37	*Sinocyclocheilusanatirostris*	Leye County, Guangxi, China	XH1901	AY854708	AY854765
38	*Sinocyclocheilusanshuiensis*	Lingyun County, Guangxi, China	–	KR069120	KR069120
39	*Sinocyclocheilusmicrophthalmus*	Lingyun County, Guangxi, China	NNNU201712001	MN145877	MN145877
40	*Sinocyclocheilusaltishoulderus*	Mashan County, Guangxi, China	–	FJ984568	FJ984568
41	*Sinocyclocheilusmashanensis*	–	GX0026-L18-12	MT373107	MW548430
42	*Sinocyclocheilusbrevibarbatus*	–	GX0064-L20-13	MT373106	MW548423
43	*Sinocyclocheilusjiuxuensis*	Jiuxu Town, Hechi City, Guangxi, China	XH8501	AY854736	AY854793
44	*Sinocyclocheilusangularis*	Baotian Town, Panzhou City, Guizhou, China*	GZNU20210322001	MZ636514	MZ636514
45	*Sinocyclocheiluszhenfengensis*	Zhexiang Town, Zhenfeng County, Guizhou, China*	GZNU20150112021	MW014317	MW014317
46	*Sinocyclocheilusbicornutus*	Xinlongchnag Town, Xingren City, Guizhou, China*	–	KX528071	KX528071
47	*Sinocyclocheiluslongicornus* sp. nov.	Hongguo Town, Panzhou City, Guizhou, China*	GZNU20210503016	MZ634123	MZ634125
48	*Sinocyclocheiluslongicornus* sp. nov.	Hongguo Town, Panzhou City, Guizhou, China*	GZNU20210503017	MZ634124	MZ634126
49	*Sinocyclocheilushyalinus*	Alugudong, Luxi County, Yunnan, China	XH4701	AY854721	AY854778
50	*Sinocyclocheilusrhinocerous*	Luoping County, Yunnan, China	–	KR069119	KR069119
51	*Sinocyclocheilusguanyangensis*	–	GX0173	MT373108	MW548426
52	*Sinocyclocheilusjii*	Gongcheng County, Guangxi, China	YNUSJ201308060038	MF100765	MF100765
53	*Sinocyclocheilushuangtianensis*	–	GX0175	MT373109	MW548428
54	*Sinocyclocheilusguilinensis*	–	GX0073-L17-2	MT373104	MW548427
55	*Carassiusauratus*	–	–	AB111951	AB111951
56	*Cyprinuscarpio*	–	–	JN105357	JN105357
57	*Garraorientalis*	–	–	JX290078	JX290078
58	*Neolissochilushexagonolepis*	–	–	KU380329	KU380329
59	*Schizothoraxyunnanensis*	–	–	KR780749	KR780749
60	*Barbusbarbus*	–	–	AB238965	AB238965
61	*Onychostomasimum*	–	–	KF021233	KF021233
62	*Pethiaticto*	–	–	AB238969	AB238969
63	*Myxocyprinusasiaticus*	–	–	AY526869	AY526869
64	*Daniorerio*	–	-–	KM244705	KM244705

### ﻿Phylogenetic analyses

We used a total of 108 mitochondrial gene sequences for molecular analyses (55 Cyt *b* sequences and 53 *ND4* sequences). Four samples of muscle tissues from *S. Sinocyclocheilusangustiporus*, *S.angularis*, and *Sinocyclocheiluslongicornus* sp. nov. were sequenced for two mitochondrial genes and 100 sequences from 45 species of *Sinocyclocheilus* were downloaded from GenBank. Following Wen et al. (2022), we selected *Carassiusauratus*, *Cyprinuscarpio*, *Garraorientalis*, *Neolissochilushexagonolepis*, *Schizothoraxyunnanensis*, *Barbusbarbus*, *Onychostomasimum*, *Pethiaticto*, *Myxocyprinusasiaticus*, and *Daniorerio* as outgroup (Table 2).

All sequences were assembled and aligned using the MUSCLE (Edgar 2004) module in MEGA 7.0 (Kumar et al. 2016) with default settings. Alignment results were checked by eye. Phylogenetic trees were constructed with both maximum likelihood (ML) and Bayesian inference (BI) methods. The ML was conducted in IQ-TREE 2.0.4 (Nguyen et al. 2015) with 2000 ultrafast bootstrap (UBP) replicates (Hoang et al. 2018) and was performed until a correlation coefficient of at least 0.99 was reached. The BI was performed in MrBayes 3.2.1 (Ronquist et al. 2012), and the best-fit model was obtained based on the Bayesian information criterion computed with PartitionFinder 2.1.1 (Lanfear et al. 2017). In this analysis, the first, second and third codons of both Cyt *b* and *ND4* genes were defined.

The analysis suggested the best partition scheme for each codon position of Cyt *b* and *ND4* genes. GTR+I+G, HKY+I+G, and TRN+I+G were selected for first, second, and third codons, respectively for both Cyt *b* and *ND4* genes. Two independent runs were conducted in BI analysis, each of which was performed for 2 × 10^7^ generations and sampled every 1000 generations. The first 25% of the samples were discarded as burn-in, resulting in a potential scale reduction factor of < 0.01. Nodes in the trees were considered well supported when Bayesian posterior probabilities (BPP) were ≥ 0.95 and the ML ultrafast bootstrap value (UBP) was ≥ 95%. Uncorrected *p*-distances (1000 replicates) based on Cyt *b* and *ND4* genes were calculated in MEGA 7.0 (Kumar et al. 2016).

### ﻿Morphological comparisons

Morphometric data were collected from 44 well-preserved specimens of *Sinocyclocheilus* (Suppl. material 1). A total of 31 measurements were recorded to the nearest 0.1 mm with digital calipers following the protocol of Zhao et al. (2006) and Zhao and Zhang (2009). The following measurements were taken:

**TL** total length (from the tip of snout to the end of the caudal-fin);

**SL** standard length (from the tip of the upper jaw to the position of the last half-centrum);

**BD** body depth (from the insertion of the dorsal fin vertically to the ventral midline);

**PL** predorsal length (from the tip of the upper jaw to the origin of the dorsal-fin);

**DFL** dorsal-fin depth (from the origin of the dorsal-fin to the tip of the longest ray);

**DBL** dorsal-fin length (from the origin to the insertion of dorsal-fin base);

**PAL** preanal length (from the tip of the upper jaw to the origin of the anal-fin);

**ABL** anal-fin base length (from the origin to the insertion of anal-fin base);

**AFL** anal-fin depth (from the origin of the anal-fin to the tip of the longest ray);

**PPTL** prepectoral length (from the tip of the upper jaw to the base of anterior pectoral-fin ray);

**PTBL** pectoral-fin base length (from the anterior to posterior end of pectoral-fin base);

**PTFL** pectoral-fin length (from the base of the first pectoral-fin ray to the tip of the longest ray);

**PPVL** prepelvic length (from the tip of the upper jaw to the base of the first pelvic-fin ray);

**PVBL** pelvic-fin base length (from the anterior to the posterior end of the pelvic-fin base);

**PVFL** pelvic-fin length (from the base of the first pelvic-fin ray to the tip of the longest ray);

**CPL** caudal peduncle length (from the anal-fin insertion to the position of the last centrum);

**CPD** caudal peduncle depth (depth at the narrowest part of the caudal peduncle);

**HL** head length (from the tip of the upper jaw to the posteriormost point of the operculum);

**HD** head depth at nape;

**HW** head width (widest distance between the two gill covers);

**SNL** snout length (from tip of snout to the anterior corner of the eye);

**ED** eye diameter (diameter of the exposed portion of the eyeball);

**IOD** interorbital distance (minimum distance between the eyes);

**IPND** prenostril distance (the tip of the upper jaw to the anterior margin of the anterior nostril);

**POND** distance between posterior nostrils (the shortest distance between posterior nostrils);

**UJL** upper jaw length (from the tip of the upper jaw (the symphysis of the premaxilla) to the corner of the mouth);

**LJL** lower jaw length (from the symphysis of the dentary to the corner of the mouth);

**MW** mouth width (the distance between the two corners of the mouth);

**RBL** rostral barbel length;

**MBL** maxillary barbel length;

**FHL** forehead horn length;

**PFPVL** distance from the pectoral-fin insertion to the ventral-fin origin; and

**PVAFL** distance from the insertion of the pelvic fin to the origin of the anal-fin.

We compared the morphological characters of the new species with literature data for 21 other species in the *S.angularis* and *S.microphthalmus* species groups (Table 3). We also examined the type and/or materials from the type-localities of *S.angularis*, *S.bicornutus*, *S.hyalinus*, *S.rhinocerous*, and *S.zhenfengensis* (Appendix 1). Principal component analyses (PCAs) of size-corrected measurements and simple bivariate scatterplots were used to explore and characterize the morphometric differences between the new species and *S.rhinocerous* and *S.hyalinus*. Mann–Whitney *U* tests were used to determine the significance of differences in morphometric characters between the new species and similar species, i.e., *S.angularis*, *S.bicornutus*, and *S.rhinocerous*. All statistical analyses were performed using SPSS 21.0 (SPSS, Inc., Chicago, IL, USA), and differences were considered statistically significant at *P* < 0.05. PCAs of morphological data were performed after logarithmic transformation and under conditions of no rotation. In addition, as reported by other researchers (Parsons and Jones 2000; Polaszek et al. 2010), canonical discriminant analysis (CDA, George and Paul 2010) was used to classify individuals into different groups, where *a priori* membership was determined based on specimens belonging to different species. All pre-processing of morphological data was performed in Microsoft Excel (Microsoft Corporation 2016).

**Table 3.** Comparison of the diagnostic features of the new species described here with those selected for the 21 species of the *S.angularis* and *S.microphthalmus* species groups within the genus *Sinocyclocheilus*. Grey shading indicates clear difference in character compared to that of *Sinocyclocheiluslongicornus* sp. nov.

Species	Horn length	Horn shape: forked (2), single (1), absent or indistinct (0)	Eyes: normal (2), reduced (1), absent (0)	Dorsal-fin rays	Pectoral-fin rays	Anal-fin rays	Pelvic-fin rays	Lateral-line scales/pores	Body scales	Gill rakers	Pelvic-fin rays reaches backward
*S.longicornus* sp. nov.	Long	1	1/0	ii, 7	ii, 13	iii, 5	i, 7	38–49	Absent	9	Tips of the pelvic-fin rays without reaches to the anus
*S.altishoulderus*	Absent	0	1	iv, 4–7	i, 16	iii, 3–5	i, 8	54–58	Body covered with thin scale	10–12	Tips of the pelvic-fin rays reaches to or beyond the anus
*S.anatirostris*	Short	1	0	iii, 8	i, 12–13	iii, 6	i, 6–8	33–42	Absent	8–12	Tips of the pelvic-fin rays without reaches to the anus
*S.angularis*	Short	1	1	iii, 7	i, 15–18	iii, 5	i, 8	37–39	Absent	7	Tips of the pelvic-fin rays without reaches to the anus
*S.anshuiensis*	Short	1	0	iii, 7	i, 11–12	ii, 5	i, 7	34–38	Body covered with thin scale	14	Tips of the pelvic-fin rays without reaches to the anus
*S.aquihornes*	Short	1	0	iii, 7	i, 9	ii, 5	i, 6	36	Absent	8	Tips of the pelvic-fin rays reaches to the anus
*S.bicornutus*	Short	2	1/0	iii, 7	i, 13–15	iii, 5	i, 7–9	36–40	Body covered with thin scale	7–9	Tips of the pelvic-fin rays reaches to the anus
*S.brevibarbatus*	Absent	0	2	iii, 7	i, 14–15	iii, 5	i, 8–9	49–51	Body covered with thin scale	8–9	Tips of the pelvic-fin rays without reaches to the anus
*S.broadihornes*	Short	1	1	iii, 6–7	i, 12–13	ii, 5	i, 5–6	35–37	Absent	4–6	Tips of the pelvic-fin rays reaches to or beyond the anus
*S.convexiforeheadus*	Short	1	0	iii, 7	i, 9	ii, 5	i, 6	/	Absent	/	Tips of the pelvic-fin rays without reaches to the anus
*S.flexuosdorsalis*	Short	1	1	iii, 8	i, 12–13	iii, 5	i, 7	37–41	Body covered with thin scale	10	Tip of the pelvic-fin beyond the anus
*S.furcodorsalis*	Short	2	0	iii, 7	i, 14–15	ii, 5	i, 7	40–46	Body covered with thin scale	8–10	Tips of the pelvic-fin rays reaches to the anus
*S.hyalinus*	Long	1	0	iii, 7	i, 12–13	iii, 5	ii, 6–7	35–37	Absent	7–9	Tips of the pelvic-fin rays reaches to the anus
*S.jiuxuensis*	Absent	0	1	iii, 7	ii, 12–14	ii, 5	i, 8	47–49	Body covered with thin scale	7–9	Tips of the pelvic-fin rays without reaches to the anus
*S.mashanensis*	Absent	0	2	iii, 7	i, 9–11	ii, 5	i, 7–8	47–50	Body covered with thin scale	7–9	Tips of the pelvic-fin rays reaches to the anus
*S.microphthalmus*	Absent	0	1	iii, 8	i, 12	iii, 5	i, 7	48–57	Absent	10–12	Tips of the pelvic-fin rays reaches to the anus
*S.rhinocerous*	Long	1	1	iii, 7	i, 12	iii, 5	i, 6	37–45	Absent	8	Tips of the pelvic-fin rays without reaches to the anus
*S.simengensis*	Short	1	2	iii, 7	i, 13–15	ii, 5	i, 7	56–57	Body covered with thin scale	9–10	Tips of the pelvic-fin rays without reaches to the anus
*S.tianlinensis*	Short	1	0	iii, 8	i, 12	iii, 5	i, 7	Absent	Absent	10	Tips of the pelvic-fin rays nearly reaches to the anus
*S.tianeensis*	Short	2	0	iii, 7	i, 9–11	ii, 5	i, 6	35–39	Body covered with thin scale	7–9	Tips of the pelvic-fin rays reaches to the anus
*S.tileihornes*	Long	2	1	iii, 7	i, 12–14	iii, 5	ii, 6–7	35–37	Absent	6–8	Tips of the pelvic-fin rays reaches to the anus or to the origin of the anal fin rays
*S.zhenfengensis*	Absent	0	2	iii, 6–7	i, 13–15	iii, 5	i, 7	36–41	Body covered with thin scale	7–9	Tips of the pelvic-fin rays nearly reaches to the anus

## ﻿Results

### ﻿Phylogenetic analyses and genetic divergence

**Figure 2.**
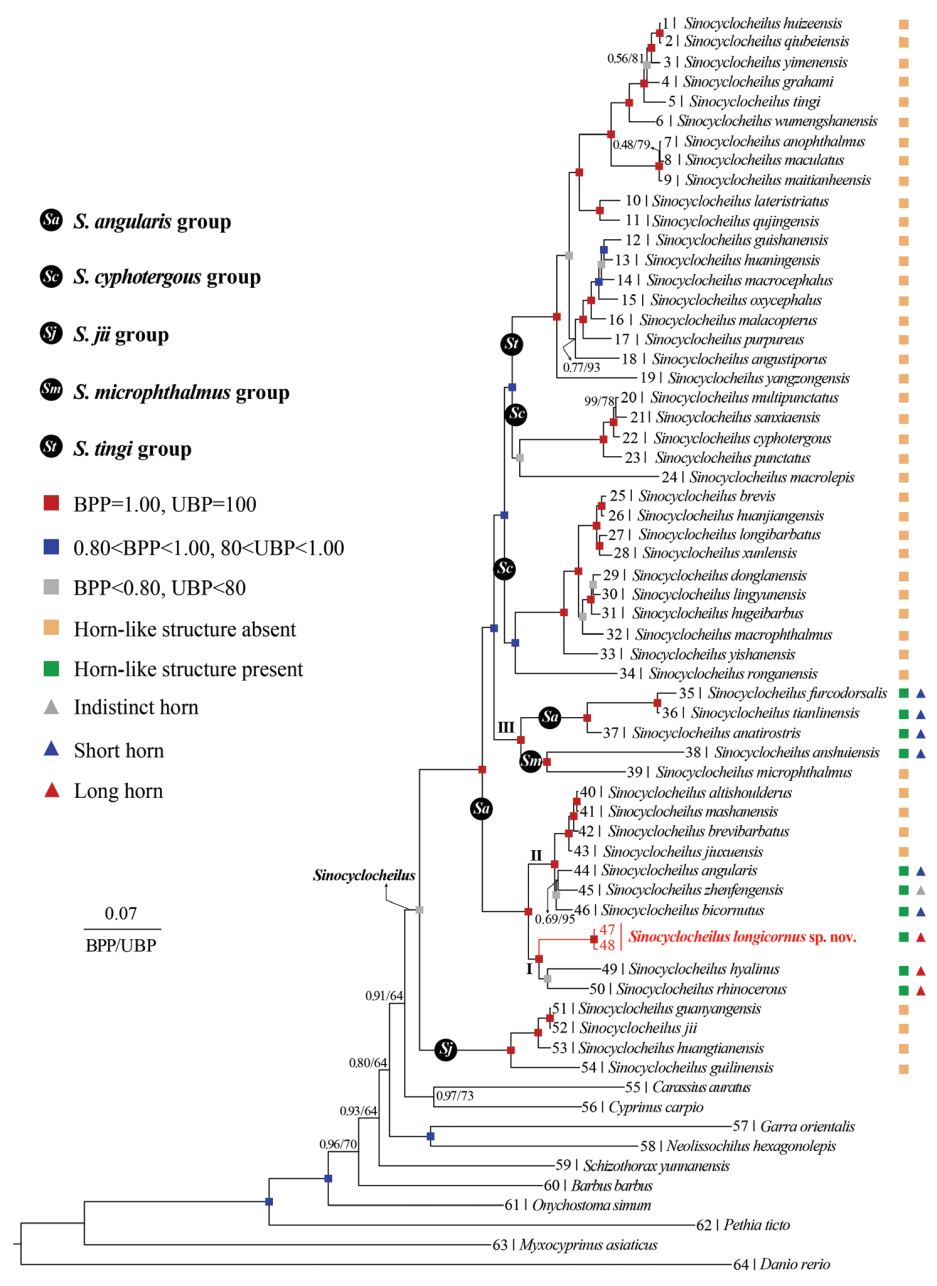
Phylogenetic tree based on mitochondrial Cyt *b* + *ND4* genes. In this phylogenetic tree, ultrafast bootstrap supports (UBP) from ML analyses/Bayesian posterior probabilities (BPP) from BI analyses were noted beside nodes. The scale bar represents 0.07 nucleotide substitutions per site. The numbers at the tip of branches corresponds to the ID numbers in Table 2. Different colored rectangular and triangular boxes in addition to the nodes denote the different states of the presence of horn-like structures of species within the genus *Sinocyclocheilus*.

ML and BI phylogenies were constructed based on two concatenated mitochondrial gene sequences, including 1140 bp Cyt *b* and 1380 bp *ND4*. The ML and the BI phylogenetic trees showed identical topology (Fig. 2). The monophyly of the genus *Sinocyclocheilus* was strongly supported by both phylogenetic analyses but the monophyly of the *S.angularis* and *S.cyphotergous* species groups was rejected (Fig. 2). In both analyses, the *S.longicornus* sp. nov. formed a highly supported clade (0.99 in BI and 96% in ML) with *S.hyalinus* and *S.rhinocerous*.

The smallest *p*-distances between *S.longicornus* sp. nov. and other species of *Sinocyclocheilus* were 6.0% in Cyt *b* (with *S.rhinocerous*) and 5.6% in *ND4* (with *S.bicornutus*). These levels of divergence were similar to those between pairs of other recognized species. For example, the Cyt *b p*-distance was 2.4% between *S.anatirostris* and *S.angularis*, 3.4% between *S.bicornutus* and *S.brevibarbatus*, while the *ND4 p*-distance was 2.7% between *S.anatirostris* and *S.angularis* and 2.6% between *S.bicornutus* and *S.anatirostris* (Suppl. materials 2, 3).

### ﻿Morphological analyses

**Figure 3.**
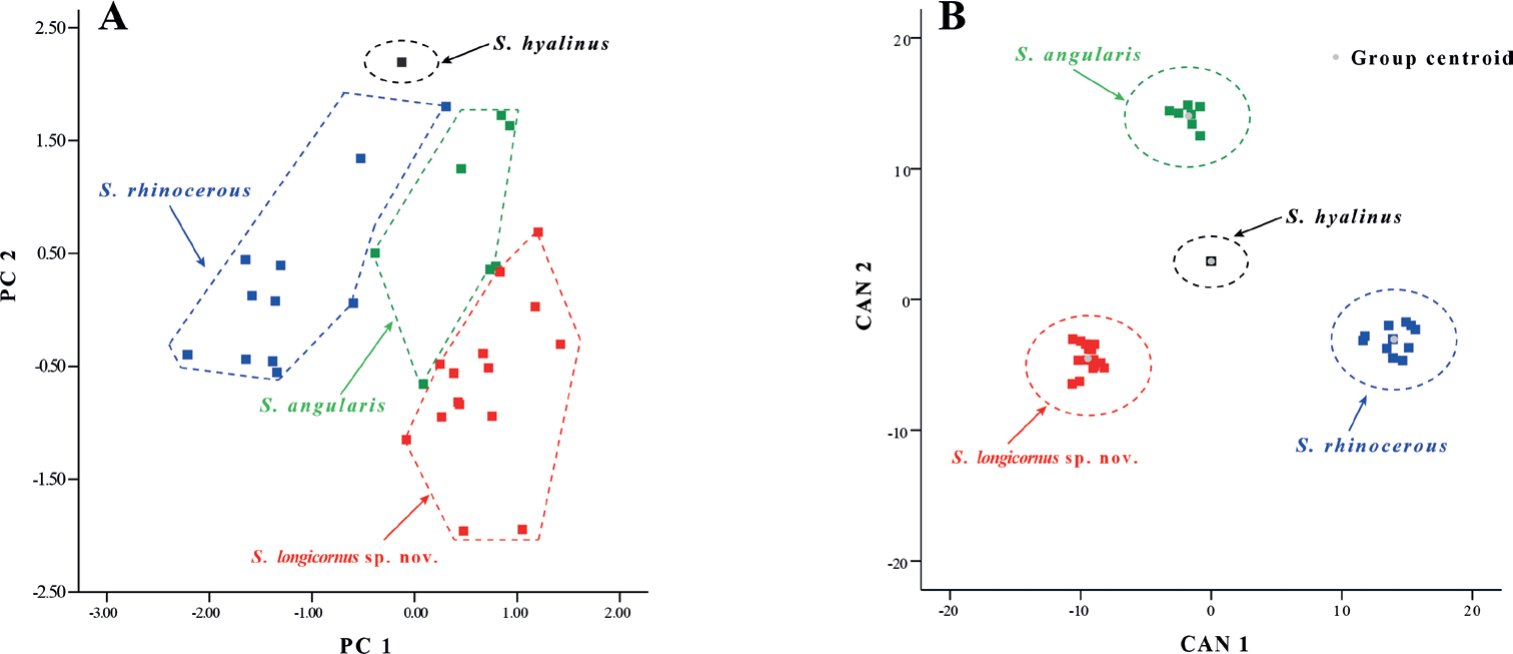
Plots of principal component analysis, and canonical discriminant analysis scores of *Sinocyclocheiluslongicornus* sp. nov., *S.angularis*, *S.rhinocerous*, and *S.hyalinus* based on morphological characters.

Mann–Whitney *U* test**s** showed that the *Sinocyclocheiluslongicornus* sp. nov. differed from *S.angularis*, *S.bicornutus*, and *S.rhinocerous* in several morphological characters (Table 4). This was specially most obvious *comparingS.longicornus* sp. nov. and *S.rhinocerous*, in wihich 87% of the morphometric characters were significantly different (*p* = 0.00–0.03) (Table 3). Based on PCA of the morphological data, two principal component factors with eigenvalues greater than two were extracted. These accounted for a total of 89.86% of the total variation (Suppl. material 4). The first principal component (PC1) accounted for 83.37% of the variation and was positively correlated with all variables (eigenvalue = 27.22), thus reflecting the morphological differences between *S.longicornus* sp. nov. and similar species. The second principal component (PC2) accounted for 4.85% of the variation and was dominated by the length of the lower jaw (LJL), length of the upper jaw (UJL), and length of the head (HL) (eigenvalue = 0.44). On the two-dimensional plots of PC1 and PC2, *S.longicornus* sp. nov. can be clearly distinguished from *S.angularis*, *S.rhinocerous*, and *S.hyalinus*, and can be almost separated from *S.angularis* (Fig. 3A). A total of 29 characters were loaded on the PC 1 axis and were mainly influenced by body length, head, and fin ray characteristics (Suppl. material 4). CDA correctly classified 100% of the individuals in the initial grouping case for the four sample groups (*N* = 36). Canonical axes (CAN) 1–3 explained 59.8%, 30.6%, and 9.6% of the total variation, respectively (Fig. 3B; Suppl. material 5). Therefore, based on PCA and CDA, the 15 specimens of *S.longicornus* sp. nov. regions in the space of morphological characters compared to four similar species.

**Table 4.** Morphological comparison of *Sinocyclocheiluslongicornus* sp. nov. (*SL*), *S.angularis* (*SA*), *S.bicornutus* (*SB*), *S.rhinocerous* (*SR*), *S.zhenfengensis* (*SZ*), and *S.hyalinus*. All units in mm. *P*-values are at 95% significance. Morphometric characters are explained in the methods section.

Measurements	*S.longicornus* sp. nov. (*N* = 15)		*S.angularis* (*N* = 7)		*S.bicornutus* (*N* = 2)		*S.rhinocerous* (*N* = 11)		*S.zhenfengensis* (*N* = 8)		*S.hyalinus* (*N* = 1)	*P*-value from Mann-Whitney U test			
	Range	Mean ± SD	Range	Mean ± SD	Range	Mean ± SD	Range	Mean ± SD	Range	Mean ± SD	Range	SL vs. SA	SL vs. SB	SL vs. SR	SL vs. SZ
TL	104.8–145.8	123.3 ± 11.3	93.8–133.1	118.7 ± 13.9	157.8–163.1	160.5 ± 3.7	60.8–107.3	76.5 ± 12.3	71.73–138.4	100.9 ± 20.9	98.9	0.731	0.015	0.000	0.013
SL	84.3–116.4	99.8 ± 9.1	76.5–106.8	96.4 ± 11.0	123.1–128.4	125.8 ± 3.7	49.1–91.1	63.2 ± 11.2	56.78–114.1	81.2 ± 17.2	80.2	0.783	0.015	0.000	0.008
BD	23.9–37.4	30.9 ± 3.7	23.8–33.5	29.2 ± 3.8	29.2–33.6	31.4 ± 3.1	11.9–23.4	16.0 ± 3.2	19.1–35.3	24.9 ± 5.6	18.8	0.581	0.824	0.000	0.008
PL	45.3–64.5	53.4 ± 5.1	41.9–58.9	51.7 ± 5.9	66.5–68.2	67.4 ± 1.2	28.5–52.2	35.4 ± 6.5	35.9–59.5	47.0 ± 7.9	47.6	0.783	0.015	0.000	0.056
DFL	12.1–17.3	14.2 ± 1.7	12.6–15.6	14.2 ± 1.3	16.3–24.1	20.2 ± 5.5	6.9–13.8	9.1 ± 1.9	8.6–16.9	11.9 ± 2.7	12.8	0.837	0.059	0.000	0.023
DBL	10.9–24.6	19.8 ± 3.0	14.7–23.2	20.4 ± 2.9	25.6–29.6	27.6 ± 2.8	10.7–17.6	14.0 ± 2.2	13.3–27.5	17.8 ± 5.0	16.6	0.447	0.015	0.000	0.115
PAL	58.1–83.6	70.6 ± 7.4	53.9–78.9	70.0 ± 9.0	89.4–93.9	91.7 ± 3.2	14.6–64.3	41.3 ± 11.9	50.1–81.6	62.5 ± 11.8	59.2	1.000	0.015	0.000	0.101
ABL	6.9–11.8	8.9 ± 1.3	7.3–9.3	8.4 ± 0.7	9.9–12.2	11.1 ± 1.6	4.2–9.3	6.0 ± 1.5	5.7–10.1	7.7 ± 1.5	9.8	0.581	0.088	0.001	0.076
AFL	14.9–21.5	18.1 ± 1.8	12.8–17.6	15.5 ± 1.8	22.5–24.7	23.6 ± 1.6	8.6–15.9	11.5 ± 1.8	11.3–17.77	14.2 ± 2.2	13.9	0.014	0.015	0.000	0.001
PPTL	26.4–36.4	30.8 ± 2.8	22.7–33.1	29.1 ± 3.6	38.8–39.2	39.0 ± 0.3	16.6–32.8	22.4 ± 4.3	20.1–33.73	25.9 ± 5.1	29.6	0.407	0.015	0.001	0.040
PTBL	2.5–4.6	3.8 ± 0.6	3.3–5.1	4.3 ± 0.5	6.5–6.5	6.5 ± 0.0	1.5–3.3	2.2 ± 0.5	2.7–5.1	3.9 ± 0.8	2.7	0.106	0.015	0.000	0.776
PTFL	17.9–30.8	23.7 ± 3.1	15.7–22.8	20.9 ± 2.5	27.5–30.9	29.2 ± 2.4	10.9–21.2	13.5 ± 2.8	13.8–24.78	18.8 ± 4.1	18.0	0.047	0.059	0.000	0.016
PPVL	41.9–61.8	50.8 ± 5.1	38.5–56.4	50.4 ± 6.0	66.3–66.7	66.5 ± 0.3	23.6–47.7	31.9 ± 6.4	36.3–62.1	46.1 ± 9.9	46.3	0.837	0.015	0.000	0.149
PVBL	3.1–5.6	4.2 ± 0.8	3.6–5.6	4.6 ± 0.7	5.1–5.9	5.5 ± 0.6	1.7–4.5	2.7 ± 0.8	2.5–5.3	4.0 ± 1.0	2.9	0.267	0.059	0.001	0.636
PVFL	12.9–46.8	17.6 ± 8.2	12.4–15.1	14.0 ± 0.9	19.2–22.6	20.9 ± 2.4	7.1–14.3	10.3 ± 2.2	11.8–16.83	14.1 ± 2.2	11.5	0.014	0.059	0.000	0.131
CPL	13.6–25.3	21.0 ± 3.7	12.3–22	18.6 ± 3.9	20.1–22.6	21.4 ± 1.8	9.6–16.7	12.6 ± 2.2	14.5–22.01	17.6 ± 2.5	14.5	0.142	1.000	0.000	0.034
CPD	8.9–13.1	11.2 ± 1.2	8.8–13.1	11.7 ± 1.5	10.6–13.4	12.0 ± 2.0	3.7–8.3	6.1 ± 1.3	7.8–12.6	10.1 ± 1.8	5.5	0.368	0.529	0.000	0.149
HL	24.3–34.6	28.7 ± 3.1	22.7–32.1	27.6 ± 3.4	38.1–39.6	38.9 ± 1.1	16.1–30.9	20.7 ± 4.0	19.8–34.8	26.1 ± 4.8	27.7	0.680	0.015	0.001	0.190
HD	14.7–22.6	17.9 ± 2.3	12.7–17.9	15.7 ± 1.8	19.2–26.9	23.1 ± 5.4	7.9–15.6	10.6 ± 2.2	11.9–19.85	15.6 ± 2.7	16	0.047	0.176	0.000	0.040
HW	11.6–17.2	14.0 ± 1.8	10.9–14.2	13.0 ± 1.3	17.4–21.2	19.3 ± 2.7	6.7–12.4	8.6 ± 1.8	9.5–17.2	13.3 ± 2.4	11.5	0.380	0.015	0.000	0.506
SNL	10.5–15.4	12.4 ± 1.5	8.5–12.6	11.3 ± 1.5	13.4–16.1	14.8 ± 1.9	6.1–11.7	8.3 ± 1.6	7.2–14.2	9.9 ± 2.0	12.9	0.332	0.176	0.000	0.001
ED	0–1.6	1.1 ± 0.5	1.5–2.6	1.9 ± 0.4	2.2–3.5	2.9 ± 0.9	0.6–4.1	1.3 ± 1.0	2.1–2.84	2.3 ± 0.2	0	0.000	0.015	0.291	0.000
IOD	5.5–8.6	7.0 ± 0.9	7.2–8.7	7.9 ± 0.6	6.2–7.8	7.0 ± 1.1	3.2–7.4	4.3 ± 1.3	5.9–9.28	7.0 ± 1.2	/	0.056	1.000	0.000	0.591
IPND	2.9–4.7	3.9 ± 0.6	3.1–4.8	4.0 ± 0.6	4.8–7.2	6.0 ± 1.7	1.3–3.3	2.1 ± 0.7	3.8–6.9	4.8 ± 0.9	3.9	0.783	0.015	0.000	0.013
POND	4.3–6.6	5.5 ± 0.6	4.2–5.9	5.0 ± 0.6	6.5–7.1	6.8 ± 0.4	3.1–5.9	4.1 ± 0.8	3.9–6.66	5.1 ± 1.1	/	0.106	0.029	0.000	0.213
UJL	4.2–6.9	5.3 ± 0.7	4.8–7.6	6.2 ± 1.2	8.5–8.5	8.5 ± 0.0	3.5–6.2	4.6 ± 0.8	3.8–7.27	5.4 ± 1.1	6.7	0.123	0.015	0.028	0.925
LJL	3.2–5.6	4.5 ± 0.6	3.9–6.3	5.3 ± 0.9	7.5–7.6	7.6 ± 0.1	3.4–6.2	4.4 ± 0.8	3.3–5.6	4.6 ± 0.9	5.1	0.039	0.015	0.367	0.825
MW	4.6–7.9	6.1 ± 1.0	4.2–7.4	6.1 ± 1.2	6.8–9.7	8.3 ± 2.1	2.5–5.8	3.6 ± 1.0	4.5–8.49	6.8 ± 1.3	5.9	1.000	0.176	0.000	0.169
RBL	10.8–19.7	15.5 ± 2.5	7.8–15.1	12.2 ± 2.4	21.3–22.2	21.8 ± 0.6	2.9–7.9	5.9 ± 1.8	8.9–15.3	11.9 ± 2.2	3.8	0.011	0.015	0.000	0.003
MBL	10.1–18.3	14.2 ± 2.5	7.2–14.7	11.1 ± 2.5	22.4–23.1	22.8 ± 0.5	2.6–7.9	6.0 ± 1.9	8.5–14.1	11.9 ± 1.7	3.3	0.014	0.015	0.000	0.034
FHL	12.5–18.2	14.7 ± 1.5	8.4–13.4	10.6 ± 1.9	13.2–13.3	13.3 ± 0.1	7.0–13.6	9.4 ± 2.1	0–0	0.0 ± 0.0	12.9	0.000	0.059	0.000	0.000
PFPVL	15.3–24.5	19.1 ± 2.3	14.3–22.8	18.6 ± 3.0	21.3–22.6	22.0 ± 0.9	7.1–14.3	9.5 ± 2.2	13.7–28.6	18.1 ± 4.9	12.4	0.680	0.088	0.000	0.190
PVAFL	13.2–22.9	18.6 ± 2.8	14.1–22.8	18.8 ± 3.0	21.8–23.3	22.6 ± 1.1	7.0–14.3	9.8 ± 2.1	12.4–19.9	15.2 ± 2.7	12.6	0.891	0.059	0.000	0.013
SL/TL	0.79–0.83	0.81 ± 0.01	0.80–0.83	0.81 ± 0.01	0.78–0.79	0.78 ± 0.01	0.80–0.85	0.82 ± 0.02	0.79–0.82	0.80 ± 0.01	0.81	0.332	0.015	0.266	0.325
SL/BD	2.98–3.66	3.24 ± 0.19	3.18–3.60	3.31 ± 0.15	3.82–4.22	4.02 ± 0.28	3.76–4.59	3.96 ± 0.24	1.95–3.97	3.34 ± 0.61	4.27	0.267	0.015	0.000	0.056
SL/HL	3.12–3.70	3.49 ± 0.14	3.33–3.72	3.50 ± 0.14	3.23–3.24	3.24 ± 0.01	2.93–3.17	3.06 ± 0.08	1.86–3.52	3.14 ± 0.53	2.90	0.945	0.059	0.000	0.003
SL/CPL	4.18–6.72	4.85 ± 0.69	4.83–6.66	5.31 ± 0.78	5.68–6.12	5.90 ± 0.31	4.28–5.72	5.05 ± 0.50	2.58–5.88	4.67 ± 0.99	5.53	0.032	0.088	0.238	0.776
SL/CPD	8.04–9.84	8.95 ± 0.63	7.69–8.69	8.25 ± 0.36	9.58–11.61	10.60 ± 1.44	8.62–13.27	10.61 ± 1.42	4.59–9.15	8.14 ± 1.49	14.58	0.056	0.059	0.003	0.131
SL/PL	1.77–2.00	1.87 ± 0.06	1.78–1.98	1.87 ± 0.07	1.85–1.88	1.87 ± 0.02	1.72–1.87	1.79 ± 0.04	1.06–1.94	1.74 ± 0.28	1.68	0.783	1.000	0.001	0.213
SL/PPTL	3.06–3.46	3.25 ± 0.12	3.19–3.43	3.32 ± 0.09	3.14–3.31	3.22 ± 0.12	2.77–2.96	2.83 ± 0.06	1.68–3.80	3.20 ± 0.64	2.71	0.185	1.000	0.000	0.169
SL/PPVL	1.87–2.06	1.97 ± 0.05	1.83–1.99	1.92 ± 0.06	1.86–1.93	1.89 ± 0.05	1.91–2.08	1.99 ± 0.05	0.96–1.97	1.80 ± 0.34	1.73	0.066	0.088	0.184	0.028
SL/PAL	1.36–1.48	1.42 ± 0.04	1.32–1.42	1.38 ± 0.03	1.31–1.44	1.37 ± 0.09	1.37–3.99	1.68 ± 0.77	0.74–1.46	1.32 ± 0.23	1.35	0.106	0.441	0.023	0.238
CPL/CPD	1.25–2.35	1.88 ± 0.27	1.15–1.78	1.58 ± 0.22	1.69–1.90	1.79 ± 0.15	1.72–2.71	2.11 ± 0.30	1.45–1.89	1.76 ± 0.17	2.64	0.011	0.618	0.066	0.149
HL/SNL	2.04–2.55	2.32 ± 0.13	2.25–2.67	2.45 ± 0.14	2.46–2.84	2.65 ± 0.27	2.31–2.78	2.51 ± 0.15	2.27–3.54	2.67 ± 0.39	2.15	0.066	0.059	0.008	0.002
HL/HW	1.79–2.34	2.06 ± 0.14	2.04–2.26	2.11 ± 0.10	1.87–2.19	2.03 ± 0.23	2.19–2.67	2.43 ± 0.16	1.82–2.08	1.97 ± 0.08	2.41	0.581	0.824	0.000	0.131
HL/HD	1.43–1.78	1.61 ± 0.10	1.60–1.92	1.76 ± 0.10	1.47–1.98	1.73 ± 0.36	1.77–2.16	1.96 ± 0.11	1.54–1.85	1.67 ± 0.09	1.73	0.007	0.824	0.000	0.149
HL/RBL	1.47–2.46	1.88 ± 0.27	1.88–2.91	2.30 ± 0.37	1.78–1.79	1.79 ± 0.00	2.34–5.55	3.79 ± 1.04	1.90–2.41	2.20 ± 0.18	7.29	0.017	0.529	0.000	0.007
HL/MBL	1.77–2.75	2.05 ± 0.26	1.93–3.15	2.56 ± 0.47	1.65–1.77	1.71 ± 0.08	2.31–6.19	3.82 ± 1.32	1.92–2.59	2.20 ± 0.24	8.39	0.007	0.015	0.000	0.131
HL/IPND	6.20–9.59	7.37 ± 0.88	5.97–10.35	7.06 ± 1.51	5.50–7.94	6.72 ± 1.72	5.73–14.77	10.45 ± 2.55	4.98–6.51	5.47 ± 0.53	7.10	0.210	0.529	0.012	0.000
HL/POND	2.07–3.06	2.56 ± 0.25	2.41–2.96	2.63 ± 0.21	2.45–3.26	2.86 ± 0.57	1.84–2.45	2.10 ± 0.19	2.39–2.88	2.65 ± 0.20	/	0.630	0.618	0.000	0.466
PTFL/PFPVL	1.09–1.40	1.24 ± 0.08	1.00–1.27	1.13 ± 0.11	1.22–1.45	1.33 ± 0.17	1.20–1.62	1.43 ± 0.13	0.81–1.71	1.07 ± 0.29	1.45	0.066	0.368	0.003	0.007
PVFL/PVAFL	0.74–2.14	0.94 ± 0.34	0.61–0.88	0.76 ± 0.10	0.82–1.04	0.93 ± 0.15	0.72–1.40	1.06 ± 0.17	0.79–1.34	0.95 ± 0.18	0.91	0.056	0.721	0.021	0.392
HW/IOD	1.68–2.64	2.01 ± 0.29	1.51–1.86	1.66 ± 0.15	2.72–2.81	2.76 ± 0.06	1.04–2.53	2.07 ± 0.39	1.61–2.07	1.89 ± 0.16	/	0.004	0.015	0.186	0.728

### ﻿Taxonomic account

#### ﻿ Sinocyclocheilus longicornus

Taxon classificationAnimaliaCypriniformesCyprinidae

Luo, Xu, Wu, Zhou & Zhou sp. nov.

EBB22465-0664-5390-A7B7-7ABFD5F242E3

https://zoobank.org/F447A6B3-1304-4734-BC57-B46E32034451

**Figure 4.**
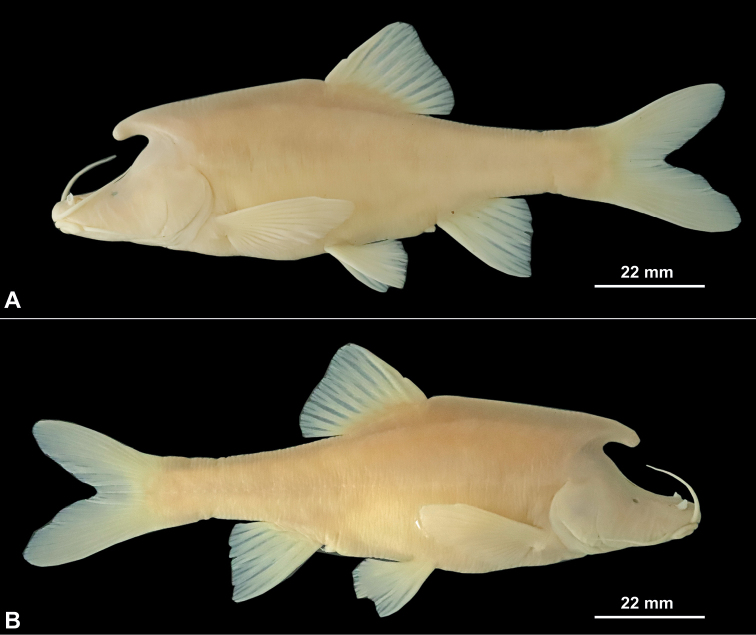
Lateral view of adult male holotype GZNU20210503002 of *Sinocyclocheiluslongicornus* sp. nov. in preservative. **A** left side view **B** right side view.

**Figure 5.**
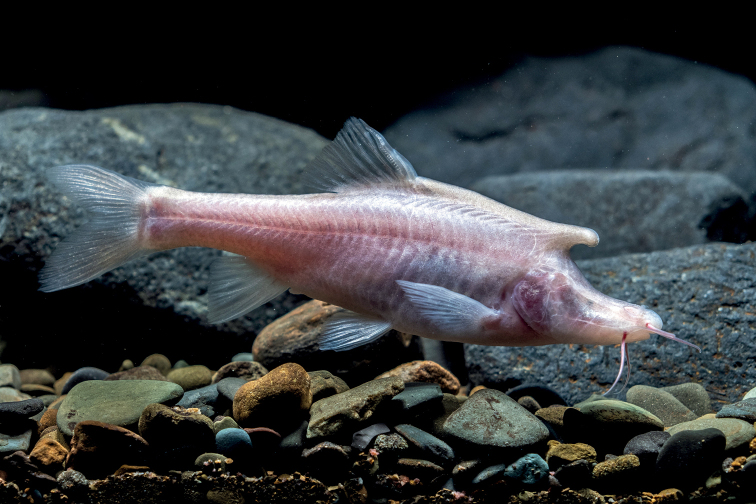
Live adult male paratype of *Sinocyclocheiluslongicornus* sp. nov.

Figs 4 , 5 , Suppl. material 1

##### Material examined.

***Holotype*.** GZNU20210503002, 135.9 mm total length (TL), 109.8 mm standard length (SL), adult male collected by Jia-Jun Zhou and Tao Luo on May 6, 2021 in Hongguo Town, Panzhou City, Guizhou Province, China (25.6576°N, 104.4044°E; ca. 1852 m a.s.l.). ***Paratypes*.** Fourteen adult male specimens from the same locality as the holotype: GZNU20210503001, GZNU20210503003–03013, GZNU20210503015–503016, 84.3–116.4 mm SL, collected by Tao Luo, Jia-Jun Zhou, and Xing-Liang Wang on May 6, 2021.

##### Diagnosis.

*Sinocyclocheiluslongicornus* sp. nov. can be distinguished from all other congeners by the following combination of characters: (1) having a single, relatively long horn-like structure on the back of the head; (2) body scaleless, albinotic body without pigmentation; (3) reduced eyes; (4) dorsal-fin rays, ii, 7; (5) pectoral-fin rays, i, 13; (6) anal-fin rays, iii, 5; (7) pelvic-fin rays, i, 7; (8) lateral line pores 38–49; (9) gill rakers well developed, 9 on first gill arch; (10) tip of the pelvic-fin rays not reaching the anus when pelvic-fin rays extended backward.

##### Description.

Body moderately elongate and compressed. Dorsal profile convex from nape to dorsal-fin; greatest body depth at dorsal-fin insertion; ventral profile slightly concave, tapering gradually toward the caudal-fin; greatest body depth slightly anterior to dorsal-fin insertion.

Head short, compressed laterally, length longer than maximum head width, depth longer than maximum head width. large and long anterior horn-like structure present on back of head not forked at tip, at about 45° angle to horizontal and curved downward at tip. Reduced eyes present in upper half of head; eye diameter less than interorbital distance; interorbital distance larger than distance between posterior nostrils. Snout short, U-shaped, and projecting beyond lower jaw in dorsal view, less than half head length.

Mouth subterminal, with slightly projecting upper jaw. Two pairs of nostrils, anterior and posterior nostrils neighboring, nares at 1/3 between snout tip and anterior margin of eye; anterior nares possessing an anterior rim with a posterior fleshy flap forming a half-tube. Two pairs of barbels; rostral barbels long, insertion of rostral barbel in front of anterior nostril, not reaching anterior edge of operculum when rostral bent backward; maxillary barbel slightly shorter compared to rostral barbel, tip surpassing eye but not reaching anterior edge of operculum when bent backward. Gill opening large, opercular membranes connected at isthmus, gill rakers well developed, nine on first gill arch. Pharyngeal teeth in three rows with counts of 2, 3, 5–5, 3, 2; pharyngeal teeth strong and well developed, with curved and pointed tips.

Dorsal fin with two unbranched and seven branched rays; last unbranched dorsal-fin ray hard at base, softening toward tip, with strong serrations along posterior edge; distal margin slightly concave, origin slightly anterior to, or superior to, pelvic-fin insertion and closer to caudal-fin base than to snout tip. Pectoral fin long with one unbranched and 13 branched rays; tip of depressed fin extending about midway between pectoral fin and pelvic-fin insertion; extending from posterior to pelvic-fin insertion and reaching to 35.44% of pelvic-fin length. Pelvic-fin long with one unbranched and seven branched rays, insertion slightly in front of dorsal-fin insertion, tip of the pelvic-fin rays not reaching the anus when pelvic-fin rays extended backward. Anus closer to anal-fin insertion than pelvic-fin insertion; anal fin with three unbranched and five branched rays; tip of anal-fin not reaching to caudal-fin base. Caudal fin with 17 branched rays and 14 unbranched rays, strongly forked; upper and lower lobes broadly pointed, unequal in length and shape.

Lateral line complete, slightly straight, curved upward at the anus position, originating from posterior margin of operculum and extending to end of caudal peduncle. Body scaleless, lateral line pores 38–49.

***Coloration of holotype*.** In life, body overall white, slightly pink posterior to dorsal fin; barbels and gills red (Fig. 5); with white granular nuptial organs on dorsal surfaces of horn-like structure on back of head and snout (Fig. 5). In 10% formalin, body overall light yellow; posterior part of operculum and all fins partially transparent (Fig. 4).

##### Comparative morphology.

*Sinocyclocheiluslongicornus* sp. nov. is assigned to the *Sinocyclocheilusangularis* species group based on phylogenetic analysis and the shared presence of the anterior horn-like structure on the back of the head (Fig. 2; Zhao and Zhang 2009). Comparative data of *Sinocyclocheiluslongicornus* sp. nov. with the 21 recognized species in the *S.angularis* and *S.microphthalmus* species groups are given in Table 3.

*Sinocyclocheiluslongicornus* sp. nov. differs from 55 species in the *S.cyphotergous*, *S.jii*, and *S.tingi* species groups by the presence of a horn-like structure on the back of the head (vs. absent). From the 21 species in the *S.angularis* and *S.microphthalmus* species groups, *Sinocyclocheiluslongicornus* sp. nov. can be distinguished from *S.altishoulderus*, *S.jiuxuensis*, *S.brevibarbatus*, *S.microphthalmus*, *S.zhenfengensis*, and *S.mashanensis* by having a long horn-like structure on the back of the head (vs. absent or indistinct), further distinguished from *S.brevibarbatus*, *S.mashanensis*, *S.simengensis*, *S.zhenfengensis* by reduced eyes (vs. normal); differs from *S.furcodorsalis*, *S.hyalinus*, *S.anatirostris*, *S.aquihornes*, *S.tianlinensis*, *S.anshuiensis*, *S.convexiforeheadus*, and *S.tianeensis* by reduced eyes (vs. absent).

*Sinocyclocheiluslongicornus* sp. nov. differs from *S.angularis* by having a relatively long horn-like structure (14.7 ± 1.5 mm vs. 10.6 ± 1.9 mm; *p*-value < 0.01, Table 4), long rostral and maxillary barbels (*p*-value < 0.05, Table 4), two unbranched dorsal-fin rays (vs. three), pectoral-fin rays (ii, 13 vs. i, 15–18), pelvic-fin rays (i, 7 vs. i, 8–10), gill rakers (nine vs. seven), and body scaleless (vs. body covered with thin scales); from *S.bicornutus* by single horn-like structure on the back of the head (vs. forked), dorsal fin rays (ii, 7 vs. iii, 7), pectoral-fin rays (ii, 13 vs. i, 15–18), body scaleless (vs. body covered with thin scales), and tip of the pelvic-fin rays not reaching the anus when pelvic-fin rays extended backward (vs. beyond the anus); from *S.broadihornes* and *S.simengensis* by dorsal fin rays (ii, 7 vs. iii, 6–7), anal-fin rays (iii, 5 vs. ii, 5), and lateral line pores (38–49 vs. 35–37 in *S.broadihornes* and 56–57 in *S.simengensis*); from *S.flexuosdorsalis* by having a relatively long horn-like structure (vs. short), dorsal-fin rays (ii, 7 vs. iii, 8), pectoral fin rays (ii, 13 vs. i, 12–13), snout length to standard length ratio is small (12.4% vs.14.4%), body scaleless (vs. body covered with scales), and tip of the pelvic-fin rays not reaching the anus when pelvic-fin rays extended backward (vs. beyond the anus); from *S.tileihornesy* by dorsal-fin rays (ii, 7 vs. iii, 7), anal-fin rays (iii, 5 vs. ii, 5), pelvic-fin rays (i, 7 vs. ii, 6–7), pectoral fin rays (ii, 13 vs. i, 12–15), pelvic fin rays (i, 7 vs. i, 6), lateral line pores (38–49 vs. 35–37), gill rakers (9 vs. 6–8), and tip of the pelvic-fin rays not reaching the anus when pelvic-fin rays extended backward (vs. beyond the anus).

*Sinocyclocheiluslongihornes* can be morphologically distinguished from its close relatives *S.rhinocerous* and *S.hyalinus*. *Sinocyclocheiluslongicornus* sp. nov. differs from *S.hyalinus* by eyes small and degenerate (vs. absent), dorsal-fin rays (ii, 7 vs. iii, 7), pelvic-fin rays (i, 7 vs. ii, 6–7), lateral line pores (39–45 vs. 35–37), and tip of the pelvic-fin rays not reaching the anus when pelvic-fin rays extended backward (vs. beyond the anus). *Sinocyclocheiluslongicornus* sp. nov. differs from *S.rhinocerous* by having a large body size (123.3 ± 11.3 mm vs. 76.5 ± 12.3 mm; *p*-e = 0.00, Table 3), long horn-like structure (14.7 ± 1.5 mm vs. 9.4 ± 2.1 mm; *p* = 0.00, Table 3), dorsal-fin rays (ii, 7 vs. iii, 7), pectoral-fin rays (ii, 13 vs. i, 12), pelvic-fin rays (i, 7 vs. i, 6), gill rakers (9 vs. 8), and a relatively long, single horn-like structure on the back of the head (14.7 ± 1.5 mm vs. 9.4 ± 2.1 mm; *p* < 0.01, Table 4). In addition, except for morphological characteristics (eye diameter, mouth width) and some ratios, such as the SL to TL ratio, SL to CPL ratio, SL to PPVL ratio, and HW to IOD ratio, the remaining morphometric values and ratios of *Sinocyclocheiluslongicornus* sp. nov. are significantly greater than those of *S.rhinocerous*.

##### Geographical distribution and habitat.

*Sinocyclocheiluslongicornus* sp. nov. is only known from the type locality, a vertical cave some distance from Hongguo Town, Panzhou city, Guizhou, China at an elevation of 2276 m. There was no light inside the cave. Individuals of *S.longicornus* sp. nov. were located in a small pool ~ 25 m from the cave entrance. The pool was ~ 1.8 m wide and 80 cm deep, with a water temperature of ~ 16 °C at collection time and a water pH of 7.4. The 15 specimens collected on 3 May 2021 were all adult males. Therefore, we believe that the breeding period started from mid-April. Within this cave, *Sinocyclocheiluslongicornus* sp. nov. co-occurred with *Triplophysa* sp., and *Sinocyclocheilus* sp. Outside the cave, the arable land was farmed to produce maize, wheat, and potatoes.

##### Etymology.

The specific epithet *longicornus* is an invariable noun in apposition, derived from the Latin words *longus*, meaning long, and *cornu* or *cornus*, meaning horn of the forehead, in reference to the presence of a long horn-like structure on the forehead of the species. We propose the English common name Long-Horned Golden-lined Fish and the Chinese common name Cháng Jiǎo Jīn Xiàn Bā (长角金线鲃).

## ﻿Discussion

Morphological comparison and phylogenetic analysis support the generic assignment and and separate species status of *Sinocyclocheiluslongicornus* sp. nov. The genetic differences between the new species and its close relatives, *S.hyalinus* and *S.rhinocerous*, were greater than the known genetic distances between other species (Suppl. materials 3, 4). *Sinocyclocheiluslongicornus* sp. nov. the number of species of *Sinocyclocheilus* to 77, of which 13 species are recorded from Guizhou Province, China.

The genus *Sinocyclocheilus* is recognized as monophyletic, but there is no consensus on the classification of species groups (Zhao and Zhang 2009; Xiang 2014; Liu 2018; Mao et al. 2021, 2022; Wen et al. 2022). Initially, *Sinocyclocheilus* was divided into four species groups, *S.jii*, *S.angularis*, *S.cyphotergous*, and *S.tingi*, based on mitochondrial Cyt *b* and morphological differences (Zhao and Zhang 2009). Phylogenetic trees reconstructed using mitochondrial *ND4* and Cyt *b*, mitochondrial genome, and restriction site–associated DNA sequencing supported monophyly of the *S.jii* and *S.tingi* species groups and rejected monophyly of the *S.angularis* and *S.cyphotergous* species groups (Xiang 2014; Liu 2018; Mao et al. 2021, 2022; Wen et al. 2022; this study). These studies proposed new classification schemes, such as two new clades (Clades E and F) from Mao et al. (2022) and a new species group (*S.microphthalmus* group) from Wen et al. (2022). Inconsistent topological differences may be related to molecular marker types, number of species and evolutionary models. For example, a phylogenetic tree reconstructed by Mao et al. (2021) for 49 species of *Sinocyclocheilus* using the GTR+I+G model for both mitochondrial *ND4* and Cyt *b* rejected monophyly of the *S.cyphotergous* group. We reanalyzed their data for codon partitioning and found that the monophyly of both *S.angularis* and *S.cyphotergous* species groups was rejected. Different genes and different codons may have different evolutionary rates (Degnan and Rosenberg 2009), so the analysis may produce conflicting results when the same untested model is applied to different gene segments. Therefore, to resolve classification disagreements among species groups, the use of genomic data and a sufficient number of species is needed for future studies.

Variable or specialized morphological characters of *Sinocyclocheilus* are closely related to the orogeny producing dark cave environments (Yang et al. 2016; Mao et al. 2021, 2022; Wen et al. 2022). For example, horn-like structures (single or forked, long or short) or bulges on the back of the head, and degeneration or loss of eyes (Zhao and Zhang 2009). *Sinocyclocheiluslongicornus* sp. nov. has a relatively long, unforked horn-like structure on the forehead, and small, degenerated eyes. It clustered with eight species of the *S.angularis* species group on the phylogenetic tree and could be divided into Clade I and Clade II. (Fig. 2). Long and short/indistinct horn-like structures are present in Clade I and Clade II, respectively (Fig. 2). Based on the present study and previous phylogenetic trees (Mao et al. 2021, 2022; Wen et al. 2022), we hypothesize that the evolution of the forehead horn may have occurred in at least two independent formations, one weakening event and one loss event (Fig. 2). As for the eye, no corresponding clade was found within the *S.angularis* species group, and variable eye phenotypes were also reported within *S.bicornutus* (in press), which may be related to the reduction of eye size during evolution or to the abundance and deprivation of food resources during growth and development, as well as related gene mutations (Ma et al. 2020; Mao et al. 2021).

## Supplementary Material

XML Treatment for Sinocyclocheilus longicornus

## ﻿Appendix 1

**Specimens examined**

***Sinocyclocheilusangularis*** (*N* = 7): China: Guizhou Province: Panzhou City: Baotian Town, (type locality): GZNU 20210505001–05004, GZNU 20210505006–05007, GZNU 0505001, collected by Tao Luo, Jiajun Zhou and Xingliang Wang on 5 May 2021. These specimens are stored at the Guizhou Normal University, Yunyan District, Guiyang City, Guizhou Province. China.

***Sinocyclocheilusbicornutus*** (*N* = 2): China: Guizhou Province: Xingren City: Xiashan Town, Gaowu Village (type locality): GZNU 20210506001–06002, collected by Tao Luo, Jiajun Zhou and Xingliang Wang on 6 May 2021. These specimens are stored at the Guizhou Normal University, Yunyan District, Guiyang City, Guizhou Province, China.

***Sinocyclocheilushyalinus*** (*N* = 1): China: Yunnan Province: Luxi County: Alu Ancient Cave (type locality): KIZ 916001 (type locality). Currently preserved in Kunming Institute of Zoology, Chinese Academy of Sciences, China.

***Sinocyclocheilusrhinocerous*** (*N* = 11): China: Yunnan Province: Luoping County: Huancheng Township, Xiaomingzhai Group (type locality): FWOQB199309001–09006, collected by Weixian Li and Jinneng Tao in September 1993;Yunnan Province: Shizong County: Wulong Township, Huaga Village (topotype locality): FWOQB20180322001–22005, collected by Hongfu Yang on 22 March 2018. Currently these specimens are stored by Yang Hongfu at the fisheries workstation in Qubei County, Yunnan Province, China.

***Sinocyclocheiluszhenfengensis*** (*N* = 8): China: Guizhou Province: Zhenfeng County: Zhexiang Town, Shuangrufeng Scenic Area (type locality): GZNU20120701001(Holotype), GZNU20190707001–07003, GZNU20210619001–19004. These specimens are stored at the Guizhou Normal University, Yunyan District, Guiyang City, Guizhou Province, China.

## Supplementary materials

Supplementary material 1 Measurements of the adult specimens of *Sinocyclocheiluslongicornus* sp. nov. Data type table (word document). https://binary.pensoft.net/file/796517This dataset is made available under the Open Database License (http://opendatacommons.org/licenses/odbl/1.0/). The Open Database License (ODbL) is a license agreement intended to allow users to freely share, modify, and use this Dataset while maintaining this same freedom for others, provided that the original source and author(s) are credited.Cheng Xu, Tao Luo, Jia-Jun Zhou, Li Wu, Xin-Rui Zhao, Hong-Fu Yang, Ning Xiao, Jiang Zhou

Supplementary material 2 Uncorrected *p*-distance (%) between 53 species of the genus *Sinocyclocheilus* based on mitochondrial *Cyt b* sequences Data type table (excel document). https://binary.pensoft.net/file/796518This dataset is made available under the Open Database License (http://opendatacommons.org/licenses/odbl/1.0/). The Open Database License (ODbL) is a license agreement intended to allow users to freely share, modify, and use this Dataset while maintaining this same freedom for others, provided that the original source and author(s) are credited.Cheng Xu, Tao Luo, Jia-Jun Zhou, Li Wu, Xin-Rui Zhao, Hong-Fu Yang, Ning Xiao, Jiang Zhou

Supplementary material 3 Uncorrected *p*-distance (%) between 52 species of the genus *Sinocyclocheilus* based on mitochondrial *ND4* sequences Data type table (excel document). https://binary.pensoft.net/file/796519This dataset is made available under the Open Database License (http://opendatacommons.org/licenses/odbl/1.0/). The Open Database License (ODbL) is a license agreement intended to allow users to freely share, modify, and use this Dataset while maintaining this same freedom for others, provided that the original source and author(s) are credited.Cheng Xu, Tao Luo, Jia-Jun Zhou, Li Wu, Xin-Rui Zhao, Hong-Fu Yang, Ning Xiao, Jiang Zhou

Supplementary material 4 Variable loadings for principal components with Eigenvalues greater than 2, from morphometric characters corrected by SL Data type table (word document). https://binary.pensoft.net/file/796520This dataset is made available under the Open Database License (http://opendatacommons.org/licenses/odbl/1.0/). The Open Database License (ODbL) is a license agreement intended to allow users to freely share, modify, and use this Dataset while maintaining this same freedom for others, provided that the original source and author(s) are credited.Cheng Xu, Tao Luo, Jia-Jun Zhou, Li Wu, Xin-Rui Zhao, Hong-Fu Yang, Ning Xiao, Jiang Zhou

Supplementary material 5 Parameters in the canonical discriminant analysis (CDA) Data type table (word document). https://binary.pensoft.net/file/796521This dataset is made available under the Open Database License (http://opendatacommons.org/licenses/odbl/1.0/). The Open Database License (ODbL) is a license agreement intended to allow users to freely share, modify, and use this Dataset while maintaining this same freedom for others, provided that the original source and author(s) are credited.Cheng Xu, Tao Luo, Jia-Jun Zhou, Li Wu, Xin-Rui Zhao, Hong-Fu Yang, Ning Xiao, Jiang Zhou
