# Supplementary material for: ﻿Sinocyclocheiluslongicornus (Cypriniformes, Cyprinidae), a new species of microphthalmic hypogean fish from Guizhou, Southwest China
Source: Zookeys. 2023 Jan 17;1141:1–28. doi: 10.3897/zookeys.1141.91501 (PMC10208810; doi:10.3897/zookeys.1141.91501)
Supplement: Supplementary material 1 — Measurements of the adult specimens of Sinocyclocheiluslongicornus sp. nov. [file zookeys-1141-001_article-91501__-s001.docx]

**Table S2.** Measurements of the adult specimens of *Sinocyclocheilus longihornes* **sp. nov.** All units in mm. See abbreviations for the morphological characters in the Materials and Methods section. * For the holotype, ^#^ branched rays

| Species | Voucher | Dorsal fin | Pectoral fin | Tail fin^#^ | Anal fin | Pelvic fin | TL | SL | BD | PL | DFL | DBL | PAL | ABL | AFL | PPTL | PTBL | PTFL | PPVL | PVBL |
| --- | --- | --- | --- | --- | --- | --- | --- | --- | --- | --- | --- | --- | --- | --- | --- | --- | --- | --- | --- | --- |
| S. longihornes **sp. nov.** | GZNU20210503001 | ii, 7 | ii, 13 | 17 | iv, 4 | i, 7 | 124.1 | 100.9 | 32.7 | 54.5 | 14.9 | 18.6 | 70.7 | 8.9 | 16.5 | 30.9 | 4.2 | 25.1 | 50.3 | 4.8 |
| S. longihornes **sp. nov.** | GZNU20210503002* | ii, 7 | ii, 13 | 17 | iv, 4 | i, 7 | 135.9 | 109.8 | 34.4 | 59.8 | 17.1 | 19.6 | 76.1 | 10.9 | 20.4 | 35.1 | 4.4 | 27.8 | 53.9 | 5.6 |
| S. longihornes **sp. nov.** | GZNU20210503003 | ii, 7 | ii, 13 | 17 | iv, 4 | i, 7 | 104.8 | 84.3 | 23.9 | 45.3 | 12.1 | 10.9 | 58.1 | 7.8 | 15.3 | 26.4 | 2.8 | 17.9 | 41.9 | 3.9 |
| S. longihornes **sp. nov.** | GZNU20210503004 | ii, 7 | ii, 13 | 17 | iv, 4 | i, 7 | 115.9 | 91.7 | 29.9 | 49.1 | 13.1 | 17.9 | 66.3 | 9.1 | 17.1 | 28.3 | 3.1 | 21.1 | 45.8 | 4.3 |
| S. longihornes **sp. nov.** | GZNU20210503005 | ii, 7 | ii, 13 | 17 | iv, 4 | i, 7 | 138.1 | 111.5 | 37.4 | 60.5 | 16.3 | 20.8 | 80.6 | 10.4 | 18.9 | 34.4 | 4.6 | 26.4 | 57.3 | 4.5 |
| S. longihornes **sp. nov.** | GZNU20210503006 | ii, 7 | ii, 13 | 17 | iv, 4 | i, 7 | 132.1 | 107.1 | 33.3 | 56.9 | 14.5 | 20.2 | 78.2 | 8.9 | 18.8 | 32.8 | 4.1 | 24.6 | 55.1 | 4.9 |
| S. longihornes **sp. nov.** | GZNU20210503007 | ii, 7 | ii, 13 | 17 | iv, 4 | i, 7 | 145.8 | 116.4 | 36.8 | 64.5 | 17.3 | 23.3 | 83.6 | 11.8 | 21.5 | 36.4 | 4.5 | 30.8 | 61.8 | 5.5 |
| S. longihornes **sp. nov.** | GZNU20210503008 | ii, 7 | ii, 13 | 17 | iv, 4 | i, 7 | 126.8 | 102.8 | 30.9 | 51.5 | 13.8 | 20.7 | 73.7 | 9.4 | 19.2 | 29.7 | 3.8 | 23.9 | 51.5 | 3.7 |
| S. longihornes **sp. nov.** | GZNU20210503009 | ii, 7 | ii, 13 | 17 | iv, 4 | i, 7 | 126.6 | 101.4 | 32.8 | 52.8 | 15.1 | 21.5 | 71.8 | 8.7 | 19.7 | 30.3 | 4.4 | 23.9 | 52.8 | 4.7 |
| S. longihornes **sp. nov.** | GZNU20210503010 | ii, 7 | ii, 13 | 17 | iv, 4 | i, 7 | 120.6 | 99.8 | 27.3 | 52.5 | 13.8 | 24.6 | 69.4 | 9.1 | 17.9 | 30.6 | 3.2 | 22.2 | 51.5 | 3.4 |
| S. longihornes **sp. nov.** | GZNU20210503011 | ii, 7 | ii, 13 | 17 | iv, 4 | i, 7 | 114.1 | 91.5 | 28.3 | 50.2 | 12.6 | 19.8 | 65.4 | 7.5 | 18.3 | 29.9 | 3.6 | 21.1 | 48.9 | 3.3 |
| S. longihornes **sp. nov.** | GZNU20210503012 | ii, 7 | ii, 13 | 17 | iv, 4 | i, 7 | 112.4 | 91.4 | 27.7 | 51.6 | 12.3 | 20.2 | 61.8 | 6.9 | 16.9 | 29.9 | 3.9 | 23.1 | 46.4 | 3.3 |
| S. longihornes **sp. nov.** | GZNU20210503013 | ii, 7 | ii, 13 | 17 | iv, 4 | i, 7 | 111.7 | 90.4 | 28.4 | 48.5 | 12.9 | 19.2 | 62.9 | 7.9 | 16.8 | 28.3 | 2.5 | 20.6 | 46.5 | 3.8 |
| S. longihornes **sp. nov.** | GZNU20210503015 | ii, 7 | ii, 13 | 17 | iv, 4 | i, 7 | 125.9 | 103.3 | 29.8 | 53.6 | 13.4 | 20.7 | 75.8 | 8.1 | 18.6 | 31.2 | 3.7 | 24.1 | 52.9 | 4.2 |
| S. longihornes **sp. nov.** | GZNU20210503016 | ii, 7 | ii, 13 | 17 | iv, 4 | i, 7 | 115.1 | 95.2 | 30.4 | 49.3 | 13.3 | 19.1 | 64.4 | 7.7 | 14.9 | 27.5 | 3.7 | 22.3 | 46.1 | 3.1 |
| *S. angularis* | GZNU20210505001 | iii, 7 | i, 15 | 18 | iii, 5 | i, 8 | 127.2 | 103.9 | 32.7 | 54.1 | 15.4 | 22.7 | 78.9 | 8.9 | 15.7 | 30.6 | 4.1 | 22.8 | 54.4 | 4.8 |
| *S. angularis* | GZNU20210505002 | iii, 7 | i, 15 | 19 | iii, 5 | i, 8 | 133.1 | 106.8 | 33.5 | 58.9 | 15.6 | 23.2 | 78.2 | 9.3 | 17.2 | 33.1 | 4.3 | 22.5 | 56.4 | 4.8 |
| *S. angularis* | GZNU20210505003 | iii, 7 | i, 15 | 19 | iii, 5 | i, 8 | 124.4 | 101.2 | 30.9 | 51.2 | 15.6 | 20.1 | 72.2 | 8.7 | 16.9 | 29.5 | 5.1 | 22.7 | 50.9 | 5.6 |
| *S. angularis* | GZNU20210505004 | iii, 7 | i, 15 | 18 | iii, 6 | i, 8 | 129.1 | 103.9 | 30.5 | 56.3 | 14.3 | 22.1 | 74.8 | 8.7 | 17.6 | 32.6 | 4.5 | 21.8 | 54.5 | 5.1 |
| *S. angularis* | GZNU20210505005 | iii, 7 | i, 15 | 18 | iii, 6 | i, 8 | 108.2 | 87.5 | 24.3 | 45.9 | 12.9 | 20.8 | 62.5 | 8.1 | 14.4 | 26.7 | 4.3 | 20.4 | 47.7 | 3.7 |
| *S. angularis* | GZNU20210505006 | iii, 7 | i, 15 | 18 | iii, 6 | i, 8 | 114.8 | 95.3 | 28.6 | 53.4 | 13.2 | 19.3 | 69.2 | 8.1 | 14.2 | 28.4 | 4.4 | 20.1 | 50.5 | 4.6 |
| *S. angularis* | GZNU20210505007 | iii, 7 | i, 15 | 19 | iii, 6 | i, 8 | 93.8 | 76.5 | 23.8 | 41.9 | 12.6 | 14.7 | 53.9 | 7.3 | 12.8 | 22.7 | 3.3 | 15.7 | 38.5 | 3.6 |
| *S. bicornutus* | GZNU20210506001 | iii, 7 | i, 16 | 19 | iii, 5 | i, 6 | 163.1 | 128.4 | 33.6 | 68.2 | 24.1 | 29.6 | 89.4 | 12.2 | 24.7 | 38.8 | 6.5 | 30.9 | 66.7 | 5.9 |
| *S. bicornutus* | GZNU20210506002 | iii, 7 | i, 16 | 19 | iii, 5 | i, 6 | 157.8 | 123.1 | 29.2 | 66.5 | 16.3 | 25.6 | 93.9 | 9.9 | 22.5 | 39.2 | 6.5 | 27.5 | 66.3 | 5.1 |
| *S. rhinocerous* | FWOQB199309001 | iii, 7 | i, 12 | 19 | iii, 5 | i, 6 | 107.3 | 91.1 | 23.4 | 52.2 | 13.8 | 16.2 | 64.3 | 9.3 | 15.9 | 32.8 | 3.3 | 21.2 | 47.7 | 4.5 |
| *S. rhinocerous* | FWOQB199309002 | iii, 7 | i, 12 | 19 | iii, 5 | i, 6 | 72.3 | 59.8 | 15.6 | 33.5 | 8.9 | 14.2 | 39.9 | 6.1 | 11.2 | 20.7 | 2.1 | 12.5 | 29.9 | 1.9 |
| *S. rhinocerous* | FWOQB199309003 | iii, 7 | i, 12 | 19 | iii, 5 | i, 6 | 84.9 | 71.8 | 18.8 | 39.6 | 9.6 | 15.8 | 48.5 | 7.6 | 12.3 | 25.9 | 2.7 | 15.4 | 36.2 | 3.6 |
| *S. rhinocerous* | FWOQB199309004 | iii, 7 | i, 12 | 19 | iii, 5 | i, 6 | 83.6 | 70.7 | 18.8 | 38.7 | 11.3 | 17.6 | 47.6 | 7.1 | 12.8 | 25.2 | 2.5 | 13.3 | 36.8 | 2.8 |
| *S. rhinocerous* | FWOQB199309005 | iii, 7 | i, 12 | 19 | iii, 5 | i, 6 | 68.5 | 58.3 | 12.7 | 33.4 | 8.3 | 13.5 | 14.6 | 6.9 | 10.2 | 20.9 | 1.8 | 11.7 | 28.5 | 3.1 |
| *S. rhinocerous* | FWOQB199309006 | iii, 7 | i, 12 | 18 | iii, 5 | i, 6 | 60.8 | 49.1 | 11.9 | 28.5 | 7.7 | 10.7 | 33.2 | 4.9 | 8.6 | 16.6 | 1.7 | 10.9 | 23.6 | 2.2 |
| *S. rhinocerous* | FWOWB20180322001 | iii, 7 | i, 12 | 19 | iii, 5 | i, 6 | 73.9 | 59.5 | 15.5 | 31.9 | 8.2 | 14.4 | 40.8 | 5.2 | 10.9 | 20.8 | 2.5 | 13.8 | 29.2 | 2.2 |
| *S. rhinocerous* | FWOWB20180322002 | iii, 7 | i, 12 | 19 | iii, 5 | i, 6 | 75.4 | 60.3 | 15.3 | 34.1 | 7.9 | 13.2 | 41.2 | 5.3 | 11.2 | 20.9 | 1.9 | 12.7 | 30.9 | 2.2 |
| *S. rhinocerous* | FWOWB20180322003 | iii, 7 | i, 12 | 18 | iii, 5 | i, 6 | 70.6 | 58.1 | 15.2 | 32.4 | 6.9 | 11.7 | 40.9 | 4.8 | 12.1 | 20.9 | 1.5 | 12.3 | 29.5 | 1.7 |
| *S. rhinocerous* | FWOWB20180322004 | iii, 7 | i, 12 | 18 | iii, 5 | i, 6 | 77.2 | 61.6 | 15.1 | 34.5 | 8.8 | 15.6 | 45.1 | 4.9 | 10.8 | 22.1 | 1.9 | 13.1 | 31.7 | 2.5 |
| *S. rhinocerous* | FWOWB20180322005 | iii, 7 | i, 12 | 19 | iii, 5 | i, 6 | 67.5 | 54.9 | 14.1 | 30.2 | 8.3 | 11.1 | 38.4 | 4.2 | 10.8 | 19.3 | 2.1 | 11.8 | 27.3 | 2.6 |
| *S. hyalinus* | KIZ 916001 | iii, 5 | i,11 | 17 | iii, 5 | i, 7 | 98.9 | 80.2 | 18.8 | 47.6 | 12.8 | 16.6 | 59.2 | 9.8 | 13.9 | 29.6 | 2.7 | 18 | 46.3 | 2.9 |
| *S. zhengfengensis* | GZNU20120701001 | iii, 7 | i, 13 | 17 | iii, 5 | i, 7 | 71.73 | 56.78 | 29.09 | 53.43 | 12.26 | 22.75 | 76.58 | 8.82 | 17.77 | 33.73 | 4.68 | 24.78 | 59.26 | 5.3 |
| *S. zhengfengensis* | GZNU20190707001 | iii, 7 | i, 15 | 17 | iii, 5 | i, 7 | 138.4 | 114.1 | 35.3 | 59.5 | 16.9 | 27.5 | 81.6 | 10.1 | 16.5 | 32.9 | 5.1 | 23.1 | 62.1 | 5.3 |
| *S. zhengfengensis* | GZNU20190707002 | iii, 6 | i, 15 | 17 | iii, 5 | i, 7 | 112.4 | 88.6 | 26.3 | 51.3 | 12.4 | 16.8 | 65.2 | 8.5 | 14.8 | 23.3 | 4.3 | 17.4 | 46.4 | 4.2 |
| *S. zhengfengensis* | GZNU20190707003 | iii, 6 | i, 15 | 17 | iii, 5 | i, 7 | 85.4 | 69.7 | 20.95 | 35.9 | 11.7 | 14.85 | 50.6 | 6.4 | 11.3 | 20.1 | 3.7 | 13.8 | 36.3 | 2.5 |
| *S. zhengfengensis* | GZNU20210619001 | iii, 7 | i, 15 | 17 | iii, 5 | i, 7 | 116.6 | 91.9 | 27.1 | 50.1 | 14.1 | 18.1 | 64.9 | 8.5 | 15.3 | 28.4 | 3.9 | 22.7 | 48.4 | 4.4 |
| *S. zhengfengensis* | GZNU20210619002 | iii, 7 | i, 15 | 17 | iii, 5 | i, 7 | 98.8 | 79.6 | 21.4 | 44.3 | 9.7 | 14.3 | 56.5 | 7.3 | 12.5 | 23.7 | 3.3 | 16.6 | 40.5 | 4.1 |
| *S. zhengfengensis* | GZNU20210619003 | iii, 7 | i, 15 | 17 | iii, 5 | i, 7 | 93.7 | 75.9 | 19.1 | 40.3 | 9.9 | 14.4 | 54.5 | 6.3 | 12.7 | 22.6 | 3.3 | 16.5 | 39.1 | 3.3 |
| *S. zhengfengensis* | GZNU20210619004 | iii, 7 | i, 15 | 17 | iii, 5 | i, 7 | 90.3 | 72.9 | 19.6 | 41.1 | 8.6 | 13.3 | 50.1 | 5.7 | 12.9 | 22.8 | 2.7 | 15.2 | 37.1 | 3.1 |

(Continued Table 2)

| Species | Voucher | PVFL | CPL | CPD | HL | HD | HW | SNL | EBD | IOD | IPND | POND | UJL | LJL | MW | MBL | RBL | FHL | PFPVL | PVAFL |
| --- | --- | --- | --- | --- | --- | --- | --- | --- | --- | --- | --- | --- | --- | --- | --- | --- | --- | --- | --- | --- |
| S. longihornes **sp. nov.** | GZNU20210503001 | 15.8 | 19.1 | 10.9 | 32.3 | 19.8 | 15.6 | 13.7 | 0 | 5.9 | 4.0 | 5.1 | 6.2 | 5.1 | 7.1 | 15.7 | 15.6 | 14.4 | 18.7 | 18.6 |
| S. longihornes **sp. nov.** | GZNU20210503002* | 15.8 | 23.9 | 12.3 | 32.4 | 20.2 | 15.4 | 13.9 | 1.3 | 8.4 | 4.6 | 5.9 | 6.9 | 5.6 | 7.9 | 19.7 | 18.3 | 16.1 | 19.9 | 19.8 |
| S. longihornes **sp. nov.** | GZNU20210503003 | 13.1 | 13.9 | 8.9 | 24.3 | 14.9 | 12.4 | 11.1 | 0.9 | 5.5 | 3.8 | 4.3 | 4.2 | 3.8 | 6.3 | 11.5 | 12.1 | 12.5 | 15.3 | 14.3 |
| S. longihornes **sp. nov.** | GZNU20210503004 | 14.9 | 18.3 | 11.4 | 25.8 | 17.3 | 14.4 | 10.5 | 1.0 | 6.6 | 3.6 | 5.4 | 5.2 | 4.2 | 5.9 | 17.6 | 13.6 | 13.8 | 17.7 | 17.8 |
| S. longihornes **sp. nov.** | GZNU20210503005 | 16.9 | 24.5 | 13.1 | 32.5 | 20.1 | 16.9 | 14.5 | 0 | 8.6 | 4.7 | 6.4 | 5.6 | 5.4 | 6.9 | 16.8 | 16.2 | 16.5 | 19.6 | 22.9 |
| S. longihornes **sp. nov.** | GZNU20210503006 | 46.8 | 23.3 | 12.6 | 30.5 | 19.2 | 15.6 | 13.1 | 1.6 | 7.5 | 4.5 | 5.9 | 4.6 | 4.2 | 7.2 | 15.4 | 16.1 | 16.6 | 22.5 | 21.9 |
| S. longihornes **sp. nov.** | GZNU20210503007 | 18.1 | 25.3 | 12.3 | 34.6 | 22.6 | 17.2 | 15.4 | 1.2 | 6.7 | 4.6 | 6.6 | 6.0 | 4.8 | 7.3 | 18.3 | 18.3 | 18.2 | 24.5 | 21.8 |
| S. longihornes **sp. nov.** | GZNU20210503008 | 17.9 | 22.3 | 10.9 | 27.8 | 19.4 | 12.6 | 10.9 | 1.1 | 7.1 | 2.9 | 5.6 | 6.1 | 4.6 | 5.4 | 15.9 | 12.9 | 13.7 | 20.1 | 20.0 |
| S. longihornes **sp. nov.** | GZNU20210503009 | 15.1 | 23.7 | 12.6 | 27.8 | 17.2 | 13.4 | 12.3 | 1.4 | 6.9 | 3.5 | 5.7 | 5.1 | 4.3 | 5.7 | 15.1 | 14.9 | 15.3 | 19.1 | 19.2 |
| S. longihornes **sp. nov.** | GZNU20210503010 | 15.7 | 22.4 | 10.4 | 27.8 | 16.8 | 11.9 | 13.6 | 0.9 | 6.3 | 3.5 | 5.1 | 4.3 | 3.2 | 5.6 | 12.8 | 10.1 | 13.4 | 18.5 | 17.9 |
| S. longihornes **sp. nov.** | GZNU20210503011 | 15.8 | 20.1 | 9.8 | 26.6 | 16.1 | 12.8 | 11.5 | 0.8 | 6.5 | 3.2 | 4.7 | 5.4 | 4.6 | 4.6 | 16.8 | 11.8 | 13.9 | 17.5 | 16.9 |
| S. longihornes **sp. nov.** | GZNU20210503012 | 14.1 | 13.6 | 10.9 | 26.6 | 15.5 | 12.8 | 11.9 | 1.3 | 7.1 | 3.9 | 5.2 | 4.6 | 4.0 | 5.8 | 10.8 | 10.9 | 14.1 | 18.1 | 13.2 |
| S. longihornes **sp. nov.** | GZNU20210503013 | 12.9 | 19.3 | 9.7 | 26.1 | 14.7 | 11.6 | 11.1 | 1.6 | 6.9 | 3.8 | 5.6 | 5.3 | 4.4 | 4.9 | 13.7 | 12.5 | 14.2 | 16.5 | 16.1 |
| S. longihornes **sp. nov.** | GZNU20210503015 | 15.9 | 24.7 | 10.5 | 28.5 | 16.7 | 13.6 | 11.6 | 1.4 | 7.6 | 4.6 | 5.2 | 5.3 | 4.8 | 5.8 | 15.1 | 14.8 | 13.8 | 20.9 | 20.9 |
| S. longihornes **sp. nov.** | GZNU20210503016 | 14.7 | 21.1 | 11.8 | 26.3 | 18.0 | 13.9 | 10.6 | 1.5 | 8.1 | 3.8 | 5.6 | 5.1 | 4.5 | 5.5 | 17.9 | 14.7 | 13.5 | 17.6 | 17.7 |
| *S. angularis* | GZNU20210505001 | 13.9 | 21.2 | 12.3 | 28.7 | 17.9 | 14.1 | 11.6 | 1.7 | 7.6 | 4.8 | 5.4 | 4.8 | 5.9 | 6.9 | 12.1 | 10.5 | 13.4 | 22.8 | 22.8 |
| *S. angularis* | GZNU20210505002 | 14.4 | 22.0 | 13.1 | 32.1 | 16.7 | 14.2 | 12.5 | 1.7 | 8.3 | 3.1 | 5.9 | 7.6 | 6.2 | 6.5 | 13.4 | 10.8 | 12.5 | 22.0 | 22.1 |
| *S. angularis* | GZNU20210505003 | 15.1 | 20.8 | 11.7 | 28.4 | 16.1 | 13.8 | 12.6 | 1.9 | 7.4 | 4.1 | 4.9 | 6.6 | 4.7 | 7.1 | 15.1 | 14.7 | 10.4 | 18.4 | 18.5 |
| *S. angularis* | GZNU20210505004 | 14.7 | 21.4 | 12.9 | 29.9 | 17.1 | 13.3 | 12.4 | 1.5 | 8.4 | 4.6 | 4.5 | 7.6 | 6.3 | 7.4 | 14.1 | 13.8 | 10.3 | 17.1 | 18.3 |
| *S. angularis* | GZNU20210505005 | 13.5 | 18.1 | 10.8 | 23.5 | 14.1 | 11.5 | 10.1 | 2.3 | 7.5 | 3.3 | 4.2 | 5.2 | 3.9 | 4.2 | 12.3 | 10.9 | 8.4 | 16.6 | 16.5 |
| *S. angularis* | GZNU20210505006 | 13.9 | 14.3 | 12.4 | 27.7 | 15.3 | 13.5 | 11.2 | 1.5 | 8.7 | 4.2 | 5.5 | 6.6 | 5.6 | 5.7 | 10.9 | 9.9 | 10.7 | 19.2 | 19.2 |
| *S. angularis* | GZNU20210505007 | 12.4 | 12.3 | 8.8 | 22.7 | 12.7 | 10.9 | 8.5 | 2.6 | 7.2 | 3.8 | 4.5 | 5.2 | 4.7 | 4.8 | 7.8 | 7.2 | 8.5 | 14.3 | 14.1 |
| *S. bicornutus* | GZNU20210506001 | 22.6 | 22.6 | 13.4 | 39.6 | 26.9 | 21.2 | 16.1 | 3.5 | 7.8 | 7.2 | 6.5 | 8.5 | 7.5 | 9.7 | 22.2 | 22.4 | 13.3 | 21.3 | 21.8 |
| *S. bicornutus* | GZNU20210506002 | 19.2 | 20.1 | 10.6 | 38.1 | 19.2 | 17.4 | 13.4 | 2.2 | 6.2 | 4.8 | 7.1 | 8.5 | 7.6 | 6.8 | 21.3 | 23.1 | 13.2 | 22.6 | 23.3 |
| *S. rhinocerous* | FWOQB199309001 | 14.3 | 16.7 | 8.3 | 30.9 | 15.6 | 12.4 | 11.7 | 1.6 | 5.3 | 2.9 | 5.9 | 6.2 | 6.2 | 5.8 | 7.6 | 7.1 | 13.6 | 14.3 | 14.3 |
| *S. rhinocerous* | FWOQB199309002 | 11.9 | 11.3 | 5.4 | 18.9 | 9.2 | 8.1 | 6.8 | 1.2 | 3.2 | 3.3 | 3.3 | 4.3 | 4.3 | 3.6 | 4.3 | 4.6 | 10.4 | 8.5 | 8.5 |
| *S. rhinocerous* | FWOQB199309003 | 12.5 | 12.7 | 5.9 | 23.6 | 11.9 | 10.8 | 9.3 | 1.5 | 4.6 | 2.4 | 4.7 | 4.6 | 4.5 | 3.9 | 7.7 | 7.2 | 11.9 | 12.8 | 12.3 |
| *S. rhinocerous* | FWOQB199309004 | 10.3 | 15.7 | 8.2 | 22.8 | 12.9 | 10.3 | 9.3 | 4.1 | 5.1 | 3.3 | 4.6 | 5.8 | 5.5 | 4.9 | 7.9 | 7.6 | 11.2 | 10.1 | 10.2 |
| *S. rhinocerous* | FWOQB199309005 | 10.8 | 10.2 | 5.3 | 19.9 | 9.2 | 7.7 | 7.8 | 1.3 | 7.4 | 1.6 | 3.8 | 4.2 | 3.9 | 3.3 | 3.9 | 3.9 | 8.6 | 9.6 | 10.5 |
| *S. rhinocerous* | FWOQB199309006 | 7.7 | 9.6 | 3.7 | 16.1 | 7.9 | 6.7 | 6.1 | 1.1 | 3.2 | 1.8 | 3.1 | 3.5 | 3.4 | 2.9 | 2.9 | 2.6 | 8.0 | 7.1 | 7.0 |
| *S. rhinocerous* | FWOWB20180322001 | 11.2 | 12.7 | 6.3 | 19.3 | 9.9 | 7.9 | 7.8 | 0.7 | 4.2 | 1.6 | 4.3 | 4.3 | 3.8 | 3.7 | 4.5 | 6.9 | 8.9 | 9.4 | 9.4 |
| *S. rhinocerous* | FWOWB20180322002 | 10.2 | 14.1 | 5.2 | 19.5 | 9.9 | 7.8 | 8.4 | 0.9 | 3.5 | 1.8 | 3.9 | 4.3 | 4.0 | 2.9 | 7.4 | 7.9 | 7.0 | 8.3 | 9.8 |
| *S. rhinocerous* | FWOWB20180322003 | 7.7 | 11.7 | 6.1 | 19.2 | 9.9 | 7.2 | 8.3 | 0.7 | 3.2 | 1.3 | 3.9 | 4.6 | 4.3 | 3.3 | 4.6 | 3.5 | 8.4 | 8.1 | 8.1 |
| *S. rhinocerous* | FWOWB20180322004 | 9.9 | 11.5 | 6.7 | 20.3 | 10.9 | 7.8 | 8.7 | 0.6 | 4.1 | 1.7 | 3.5 | 4.9 | 4.5 | 2.5 | 6.4 | 6.9 | 7.6 | 8.1 | 8.2 |
| *S. rhinocerous* | FWOWB20180322005 | 7.1 | 12.2 | 5.6 | 17.3 | 9.3 | 7.6 | 6.6 | 0.6 | 3.6 | 1.6 | 3.9 | 4.1 | 4.0 | 3.1 | 7.4 | 7.5 | 7.9 | 8.3 | 9.9 |
| *S. hyalinus* | KIZ 916001 | 11.5 | 14.5 | 5.5 | 27.7 | 16 | 11.5 | 12.9 | 0 | / | 3.9 | / | 6.7 | 5.1 | 5.9 | 3.8 | 3.3 | 12.9 | 12.4 | 12.6 |
| *S. zhengfengensis* | GZNU20120701001 | 16.71 | 22.01 | 12.36 | 30.59 | 19.85 | 15.9 | 8.65 | 2.84 | 9.28 | 4.7 | 6.66 | 7.27 | 5.58 | 8.49 | 12.94 | 11.8 | 0 | 14.5 | 12.5 |
| *S. zhengfengensis* | GZNU20190707001 | 16.83 | 19.4 | 12.6 | 34.8 | 18.8 | 17.2 | 14.2 | 2.4 | 8.3 | 6.9 | 6.6 | 6.6 | 5.6 | 8.2 | 15.3 | 14.1 | 0 | 28.6 | 19.9 |
| *S. zhengfengensis* | GZNU20190707002 | 14.5 | 15.8 | 10.9 | 26.4 | 15.6 | 13.6 | 10.3 | 2.2 | 6.9 | 5.2 | 5.5 | 5.5 | 5.4 | 7.7 | 11.3 | 12.7 | 0 | 20.9 | 17.8 |
| *S. zhengfengensis* | GZNU20190707003 | 11.8 | 14.5 | 7.8 | 19.8 | 11.9 | 9.5 | 7.2 | 2.1 | 5.9 | 3.8 | 3.9 | 3.8 | 3.7 | 4.5 | 8.9 | 8.5 | 0 | 16.1 | 14.9 |
| *S. zhengfengensis* | GZNU20210619001 | 16.1 | 19.6 | 10.5 | 27.9 | 16.7 | 13.8 | 10.1 | 2.3 | 6.9 | 4.7 | 4.8 | 5.6 | 5.1 | 6.7 | 14.2 | 13.5 | 0 | 19.3 | 16.1 |
| *S. zhengfengensis* | GZNU20210619002 | 12.2 | 16.4 | 8.7 | 23.6 | 14.2 | 11.8 | 10.4 | 2.1 | 6.3 | 4.3 | 4.2 | 4.7 | 3.3 | 6.3 | 12.4 | 12.3 | 0 | 15.9 | 14.7 |
| *S. zhengfengensis* | GZNU20210619003 | 12.6 | 16.7 | 9.1 | 22.9 | 14.5 | 12.6 | 9.2 | 2.3 | 6.3 | 4.6 | 4.5 | 4.9 | 4.1 | 6.4 | 10.6 | 11.6 | 0 | 15.6 | 12.9 |
| *S. zhengfengensis* | GZNU20210619004 | 12.1 | 16.6 | 8.9 | 23.1 | 13.6 | 12.1 | 9.2 | 2.4 | 6.4 | 4.2 | 4.3 | 4.9 | 4.2 | 5.9 | 9.6 | 10.8 | 0 | 13.7 | 12.4 |
