# Supplementary material for: ﻿Sinocyclocheiluslongicornus (Cypriniformes, Cyprinidae), a new species of microphthalmic hypogean fish from Guizhou, Southwest China
Source: Zookeys. 2023 Jan 17;1141:1–28. doi: 10.3897/zookeys.1141.91501 (PMC10208810; doi:10.3897/zookeys.1141.91501)
Supplement: Supplementary material 4 — Variable loadings for principal components with Eigenvalues greater than 2, from morphometric characters corrected by SL [file zookeys-1141-001_article-91501__-s004.docx]

**Table S5.** Variable loadings for principal components with Eigenvalues greater than 2, from morphometric characters corrected by SL.

| Morphometric characters | Component | |
| --- | --- | --- |
|  | PC 1 | PC 2 |
| TL | 0.994 | -0.041 |
| SL | 0.996 | -0.022 |
| BD | 0.981 | -0.083 |
| PL | 0.989 | 0.050 |
| DFL | 0.972 | 0.072 |
| DBL | 0.858 | -0.004 |
| PAL | 0.880 | -0.006 |
| ABL | 0.902 | 0.130 |
| AFL | 0.951 | -0.128 |
| PPTL | 0.968 | 0.097 |
| PTBL | 0.925 | 0.025 |
| PTFL | 0.977 | -0.097 |
| PPVL | 0.988 | 0.033 |
| PVBL | 0.889 | 0.089 |
| PVFL | 0.778 | -0.299 |
| CPL | 0.911 | -0.134 |
| CPD | 0.930 | -0.080 |
| HL | 0.968 | 0.148 |
| HD | 0.977 | -0.020 |
| HW | 0.980 | -0.010 |
| SNL | 0.935 | 0.044 |
| IPND | 0.868 | 0.032 |
| UJL | 0.678 | 0.652 |
| LJL | 0.513 | 0.779 |
| MW | 0.926 | 0.105 |
| MBL | 0.881 | -0.259 |
| RBL | 0.848 | -0.291 |
| FHL | 0.832 | -0.147 |
| PFPVL | 0.967 | -0.120 |
| PFPVL | 0.959 | -0.076 |
| Eigenvalues | 27.221 | 0.439 |
| Percentage of total variance | 83.368 | 4.847 |
| Cumulative percentage | 83.368 | 88.215 |
