# Supplementary material for: ﻿Sinocyclocheiluslongicornus (Cypriniformes, Cyprinidae), a new species of microphthalmic hypogean fish from Guizhou, Southwest China
Source: Zookeys. 2023 Jan 17;1141:1–28. doi: 10.3897/zookeys.1141.91501 (PMC10208810; doi:10.3897/zookeys.1141.91501)
Supplement: Supplementary material 5 — Parameters in the canonical discriminant analysis (CDA) [file zookeys-1141-001_article-91501__-s005.docx]

**Table S6.** Parameters in the canonical discriminant analysis (CDA).

| Morphometric characters | Canonical axis | | |
| --- | --- | --- | --- |
|  | CAN1 | CAN2 | CAN3 |
| BD | -0.181 | 0.045 | 0.085 |
| MBL | -0.180 | -0.008 | 0.141 |
| CPD | -0.172 | 0.087 | 0.14 |
| TL | -0.166 | 0.047 | 0.033 |
| SL | -0.156 | 0.046 | 0.033 |
| AFL | -0.152 | -0.012 | 0.031 |
| RBL | -0.151 | -0.014 | 0.133 |
| PTFL | -0.149 | 0.009 | 0.025 |
| PVAFL | -0.147 | 0.063 | 0.058 |
| HD | -0.146 | 0.006 | -0.019 |
| PPVL | -0.143 | 0.056 | -0.006 |
| HW | -0.139 | 0.026 | 0.015 |
| PL | -0.136 | 0.04 | 0.001 |
| PAL | -0.135 | 0.052 | 0.013 |
| DFL | -0.131 | 0.056 | -0.001 |
| IPND | -0.125 | 0.058 | -0.029 |
| PTBL | -0.122 | 0.097 | 0.058 |
| FHL | -0.12 | -0.071 | -0.028 |
| SNL | -0.115 | 0.011 | -0.056 |
| CPL | -0.11 | 0.004 | 0.041 |
| MW | -0.108 | 0.043 | -0.02 |
| PPTL | -0.103 | 0.018 | -0.021 |
| HL | -0.1 | 0.025 | -0.02 |
| ABL | -0.094 | 0.024 | -0.059 |
| DBL | -0.094 | 0.05 | 0.027 |
| PVBL | -0.087 | 0.061 | 0.054 |
| PVFL | -0.053 | -0.012 | 0.019 |
| PFPVL | -0.071 | -0.084 | 0.071 |
| UJL | -0.04 | 0.076 | -0.059 |
| LJL | -0.01 | 0.065 | -0.018 |
| Eigenvalues | 117.113 | 59.952 | 18.769 |
| Percentage of total variance | 59.8 | 30.6 | 9.6 |
| Cumulative percentage | 59.8 | 90.4 | 100 |
| Positive correlation | 0.996 | 0.992 | 0.974 |
